# Floxuridine supports UPS independent of germline signaling and proteostasis regulators via involvement of detoxification in *C*. *elegans*

**DOI:** 10.1371/journal.pgen.1011371

**Published:** 2024-07-31

**Authors:** Abhishek Anil Dubey, Anwesha Sarkar, Karolina Milcz, Natalia A. Szulc, Pankaj Thapa, Małgorzata Piechota, Remigiusz A. Serwa, Wojciech Pokrzywa

**Affiliations:** 1 Laboratory of Protein Metabolism, International Institute of Molecular and Cell Biology in Warsaw, Warsaw, Poland; 2 IMol Polish Academy of Sciences, Warsaw, Poland; University of Cologne, GERMANY

## Abstract

The ubiquitin-proteasome system (UPS) is critical for maintaining proteostasis, influencing stress resilience, lifespan, and thermal adaptability in organisms. In *Caenorhabditis elegans*, specific proteasome subunits and activators, such as RPN-6, PBS-6, and PSME-3, are associated with heat resistance, survival at cold (4°C), and enhanced longevity at moderate temperatures (15°C). Previously linked to improving proteostasis, we investigated the impact of sterility-inducing floxuridine (FUdR) on UPS functionality under proteasome dysfunction and its potential to improve cold survival. Our findings reveal that FUdR significantly enhances UPS activity and resilience during proteasome inhibition or subunit deficiency, supporting worms’ normal lifespan and adaptation to cold. Importantly, FUdR effect on UPS activity occurs independently of major proteostasis regulators and does not rely on the germ cells proliferation or spermatogenesis. Instead, FUdR activates a distinct detoxification pathway that supports UPS function, with GST-24 appearing to be one of the factors contributing to the enhanced activity of the UPS upon knockdown of the SKN-1-mediated proteasome surveillance pathway. Our study highlights FUdR unique role in the UPS modulation and its crucial contribution to enhancing survival under low-temperature stress, providing new insights into its mechanisms of action and potential therapeutic applications.

## Author summary

In our study, we investigate the ubiquitin-proteasome system (UPS) in *Caenorhabditis elegans*, a crucial cellular machinery for degrading and recycling misfolded or excess proteins. The efficiency of this system is vital for cell health, particularly under stress conditions such as cold. We found that floxuridine (FUdR), commonly used to induce sterility in nematodes, also triggers an alternative UPS-stimulating or UPS-relieving pathway. This supports worms’ lifespan and ability to survive at low temperatures (4°C) despite proteasome deficiencies. This enhancement occurs independently of major proteostasis regulators and does not rely on reproductive pathways or conventional stress response mechanisms. Instead, FUdR activates a distinct detoxification pathway that can boost UPS activity, with GST-24 appearing to be one of the contributing factors. Our findings reveal a novel aspect of FUdR action, suggesting potential strategies for enhancing cellular resilience to environmental stress and aging in broader biological contexts.

## HIGHLIGHTS

- FUdR enhances UPS activity and resilience during proteasome inhibition or subunit deficits in *C*. *elegans*, supporting normal lifespan and improving cold adaptation.
- Support of proteasome function by FUdR operates independently of well-known proteostasis regulators and does not rely on inhibiting germ cell proliferation.
- FUdR activates a detoxification pathway that augments UPS functionality, with GST-24 appearing as one of the crucial factors, especially in the context of SKN-1 depletion.

## Introduction

Maintaining protein homeostasis (proteostasis) is pivotal for cellular health, influencing longevity, metabolism, and stress resistance, as exemplified in notable studies on the nematode *Caenorhabditis elegans* [1,2,3]. A compromised proteostasis system can lead to premature aging, elevated stress sensitivity, and protein-misfolding diseases [2,3,4]. A growing body of research highlights that reproductive capacity, influenced by signals from proliferating germline stem cells (GSCs), is a critical regulator of proteostasis, impacting metabolic processes in the soma [5]. In nematodes, the GLP-1/Notch signaling pathway is crucial for regulating the GSCs pool and establishing germline polarity; moreover, mutations in GLP-1 result in germline loss [6,7]. Remarkably, *glp-1* mutant nematodes exhibit augmented stress resilience, autophagic processes, ubiquitin-proteasome system (UPS) activity, and an extended lifespan. These phenotypic changes are modulated by pivotal molecular regulators, including SKN-1, DAF-12, DAF-16, HSF-1, and target of rapamycin (TOR) [8,9,10,11,12,13,14,15].

Sterility in *C*. *elegans* can be also chemically induced using floxuridine (FUdR), a well-documented thymidylate synthase inhibitor and anti-cancer agent. FUdR disrupts DNA and RNA synthesis, causing mitotic cell death and inhibition of protein production [16]. Given that adult nematode cells are largely post-mitotic, administering FUdR just prior to sexual maturity primarily prevents progeny development without posing a major effect on the adults. While FUdR does not prolong the lifespan of wild-type worms [16], its introduction at the L4 larval stage extends the lifespans of fat-storing *tub-1* and *gas-1* mutants, which exhibit compromised mitochondrial complex functions [17,18]. Interestingly, FUdR-induced sterility in wild-type adult worms significantly enhances their proteostasis, protein folding, and stress responses [19,20,21,22]. While commonly attributed to its sterilizing capabilities, FUdR has been shown to enhance longevity and proteostasis in nematodes devoid of a germline, suggesting that its beneficial effects are not exclusively linked to reproductive inhibition [22]. It is postulated that the mechanism for FUdR proteostasis improvements stems from metabolic shifts or the activation of the DNA damage response pathway [23,24,25]. Nevertheless, the exact mechanism underlying the proteostasis improvements remains elusive.

While proteostasis has traditionally been studied in the context of heat stress, its role under cold has been less explored. Emerging evidence indicates that moderately low temperature (15°C) promotes *C*. *elegans* longevity, enhances proteostasis, and mitigates the accumulation of pathogenic proteins. This protective mechanism is driven predominantly by the PA28γ/PSME-3-activated proteasomes [26]. In parallel, Habacher and colleagues, through an RNA interference (RNAi) screen, pinpointed PBS-6, a proteasome subunit, as instrumental in cold (4°C) survival [27]. This underscores the integral role of the proteasome within the broader proteostasis context during cold adaptation. Building on this foundation, our research sought to discern the relationship between FUdR treatment and its potential role in reinforcing proteostasis, especially when confronting proteasome dysfunction with cold conditions.

Our results demonstrate that while proteasome dysfunctions hinder the nematode’s adaptation to low temperatures (4°C), treatment with FUdR significantly enhances their cold tolerance by boosting proteasome function, even in the absence of specific proteasome subunits. FUdR markedly improves the UPS activity under conditions known to impair proteasome function, such as bortezomib treatment and RNAi-mediated knockdown of proteasome subunits. Notably, the ability of FUdR to enhance proteasome activity is independent of germ cell proliferation and spermatogenesis.

Further investigations reveal that FUdR role in enhancing proteasome activity does not depend on autophagy-related pathways or established proteostasis regulators like RPN-6.1, HSF-1, and DAF-16 [20,21,28]. Our findings demonstrate that FUdR treatment enables worms to restore or stabilize UPS capabilities even when the SKN-1-mediated proteasome surveillance mechanism [29,30] is silenced, indicating the activation of a distinct proteostasis-modulating pathway. Crucially, FUdR stimulates a detoxification pathway that supports UPS activity upon SKN-1 depletion, with GST-24 playing a role in this process. These findings underscore FUdR potential for therapeutic applications in conditions characterized by proteasome impairment, providing new insights into its underlying mechanisms.

## Results

### FUdR treatment enhances proteasome activity

To study the impact of FUdR on the *in vivo* UPS activity in *C*. *elegans*, we utilized a non-cleavable ubiquitin (Ub G76V) fused to a green fluorescent protein (UbV-GFP) under the *sur-5* promoter that remains active in most tissues, as a model to assess the functioning of degradation pathway [31]. In this system, the ubiquitin moiety of the GFP substrate is poly-ubiquitinated, leading to its recognition and subsequent degradation by the 26S proteasome. In turn, disruption in the UPS activity leads to an increase in UbV-GFP levels, which can be visualized by fluorescence microscopy or western blotting [32,33]. Of note, to account for potential variation arising from the transgene expression which might influence the amount of UbV-GFP, worms were also equipped with a mCherry reporter driven by the same *sur-5* promoter [33], and the GFP fluorescent signal was normalized to the mCherry fluorescence.

**Fig 1:**
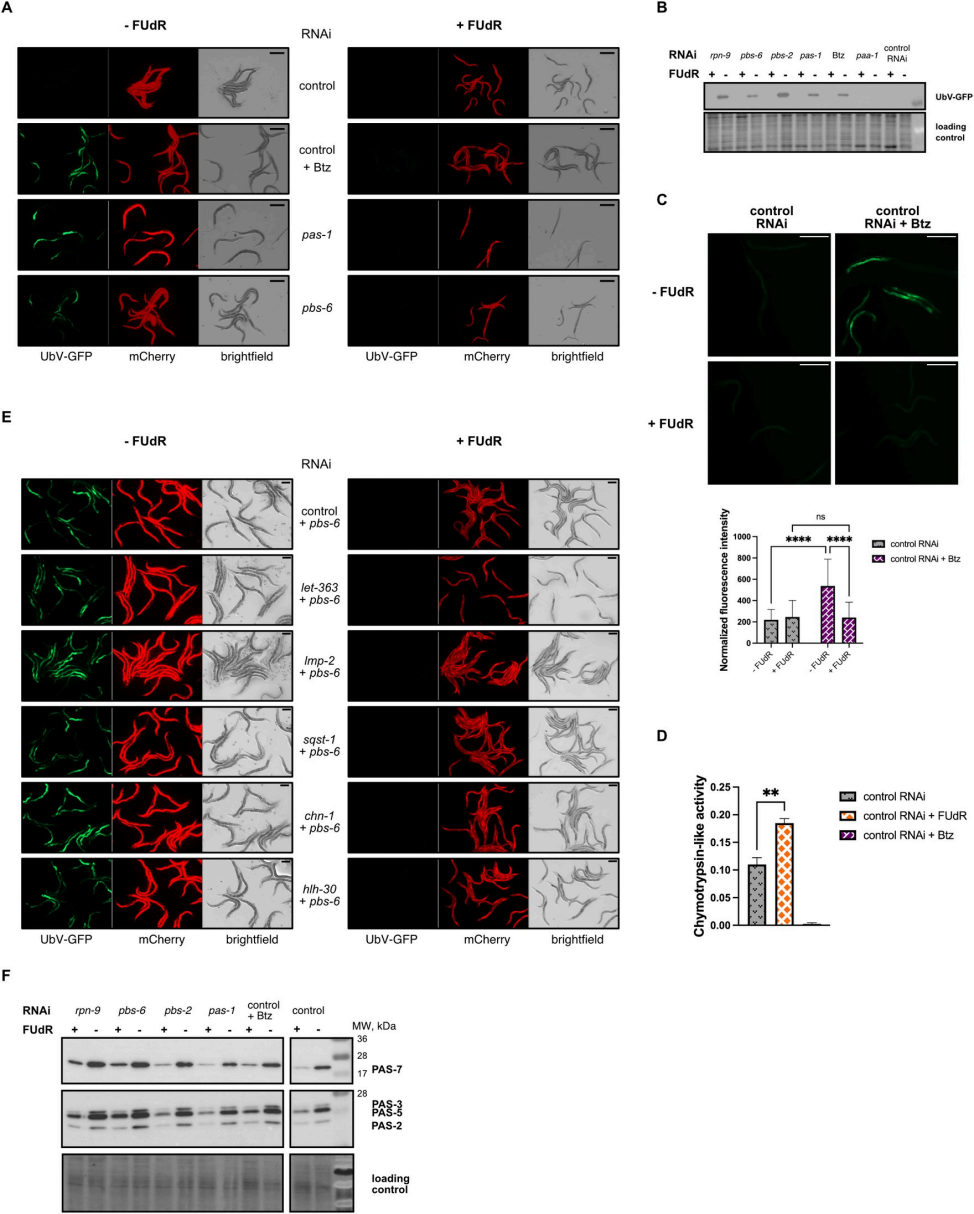
Enhancement of proteasome activity by FUdR. **(A)** FUdR impact on UbV-GFP turnover in the presence of the proteasome inhibitor bortezomib (Btz) and during the RNAi depletion of *pas-1* and *pbs-6* proteasome subunits from young adult stage. Silencing of respective genes and FUdR treatment was carried out for 40 hours. Scale bar corresponds to 400 μm. **(B)** Western blot showing the accumulation of UbV-GFP substrate in the presence of bortezomib (Btz) or either upon silencing of *paa-1*, *pas-1*, *pbs-6*, *pbs-2* and *rpn-1* from young adults, using anti-GFP antibody, in the presence and absence of FUdR. Silencing of respective genes and FUdR treatment was carried out for 40 hours. **(C)** FUdR impact on polyubiquitin reporter (*vha-6p*::*UIM2-ZsProSensor*) in the intestinal cells. Bortezomib (Btz) and FUdR treatment were initiated at the L4 stage and continued for 40 hours. Scale bar corresponds to 400μm. Below is a graph illustrating the normalized fluorescence intensity of the GFP signal across different experimental conditions. Data was analyzed using two-way ANOVA and the significance levels obtained from the Tukey’s multiple comparisons test are indicated for the compared conditions (ns—not significant, ****-P<0.0001). **(D)** FUdR effect on proteasome activity, as measured by chymotrypsin-like activity in wild-type worms with or without FUdR treatment. Bortezomib (Btz) served as the negative control. Proteasome activity is represented as slopes obtained from kinetic measurements. The experiments were conducted thrice as separate biological replicates, and significance levels (**—*P* ≤ 0.01) were determined using an unpaired t-test with Welch’s correction. **(E)** FUdR impact on UbV-GFP turnover was examined in the presence of *pbs-6* RNAi combined with either control RNAi or RNAi targeting *let-363*, *lmp-2*, *sqst-1*, *chn-1*, and *hlh-30*. FUdR treatment and gene silencing were initiated at the L4 stage and conducted for 40 hours. **(F)** Western blot showing levels of PAS-2, PAS-3, PAS-5, and PAS-7 proteasome subunits levels in wild-type worms following the RNAi depletion of RPN-9, PBS-6, PBS-2 and PAS-1 proteasome components in the presence or absence of FUdR, as depicted by using anti-proteasome 20S alpha 1+2+3+5+6+7 antibody. FUdR treatment and silencing of respective genes were carried out from the young adult stage for 40 hours. The No-Stain Protein Labeling Reagent was used to confirm equal protein loading.

Our reporter strain was exposed to distinct experimental conditions to elucidate the impact of FUdR on the UPS activity. These conditions were designed to impair proteasome function, either via the direct proteasomal inhibition with bortezomib or through RNAi targeting of proteasome subunits *pas-1* and *pbs-6*, leading to the accumulation of UbV-GFP. Intriguingly, when FUdR treatment was initiated at the young adult stage, we observed a pronounced increase in the UPS activity across all experimental conditions. This proteostasis rescue effect was evidenced by a concomitant reduction in UbV-GFP accumulation, which is attributable to accelerated GFP degradation. Conversely, worms without FUdR treatment manifested a significant accumulation of UbV-GFP owing to compromised proteasome function (Fig 1A). We validated these microscopic observations by immunoblotting protein extracts from *sur-5*∷UbV-GFP transgenic worms, treated with bortezomib or subjected to RNAi-mediated depletion of proteasomal subunits, using GFP-specific antibodies (Fig 1B). Next, we utilized a transgenic *C*. *elegans* strain expressing a fluorescent reporter designed to bind polyubiquitin chains in intestinal cells [34]. The reporter construct consists of ZsProSensor—a short-lived fluorescent protein that undergoes proteasomal degradation in an ubiquitin-independent manner—fused to ubiquitin-interacting motifs (UIMs) from the RPN-10 ubiquitin receptor. This configuration allows the reporter to bind endogenously polyubiquitinated proteins. The premise of this assay is that an increase in fluorescence intensity indicates the stabilization and accumulation of polyubiquitinated proteins, reflecting a perturbation in proteasome activity [34,35,36]. Our experimental data revealed that bortezomib treatment from L4 larval stage led to enhanced fluorescence in the reporter strain, indicative of compromised proteasome function. Furthermore, administration of FUdR significantly attenuated this fluorescence, confirming its efficacy in promoting proteasomal activity and reducing the accumulation of polyubiquitinated proteins (Fig 1C). Additionally, we conducted western blot analysis on protein extracts from *C*. *elegans* day 1 adults following RNAi-mediated knockdown of PBS-6, utilizing anti-ubiquitin antibodies. Our results demonstrated that depletion of PBS-6 led to an increase in polyubiquitinated protein levels (S1A Fig), also pointing at the impairment of proteasome function. Conversely, the application of FUdR resulted in a significant reduction of polyubiquitinated proteins (S1A Fig), corroborating its clearing effect as observed in the fluorescent reporter assays (Fig 1A).

Next, we undertook proteasome activity assays to determine if the observed FUdR-induced effects resulted from elevated proteasome activity. These employed fluorescently labeled probes [28] to measure chymotrypsin-like activity in lysates prepared from wild-type worms with or without FUdR treatment. Our data revealed that FUdR was associated with a marked increase in chymotrypsin-like activity (Fig 1D). The increase in activity does not seem to depend on the direct interaction of FUdR with the proteasome, as no changes in the processivity of the recombinant human 26S proteasome were noted in its presence (S1B Fig). We also explored the evolutionary conservation of FUdR’s impact on proteasome activity and observed its slight, non-significant increase in HeLa cells with rising FUdR levels, suggesting the full effect of FUdR might necessitate a multicellular mechanism (S1C Fig). Subsequent investigations indicated that the protective mechanism of FUdR on proteostasis does not depend on autophagy, as knockdown of autophagy-related genes *atg-1* or *lgg-1* did not impede the beneficial effects of FUdR (S1D Fig). Our studies have further extended to the domain of selective autophagy. We targeted E3 ligase CHN-1 and the aggrephagy receptor SQST-1, both of which are involved in the ubiquitination of aggrephagy substrates. Additionally, we depleted TOR kinase, encoded by *let-363*, which typically inhibits autophagy under nutrient-rich conditions. Our experiments also included the knockdown of LMP-2, which plays a role in the integrity of the lysosomal membrane, and HLH-30, a transcription factor that regulates several autophagy-related and lysosomal genes, influencing autophagic processes in *C*. *elegans* [37,38,39,40,41]. Despite these interventions, none of these knockdowns were necessary for the effect of FUdR on proteasome substrate turnover in animals with disrupted PBS-6 function (Fig 1E).

Next, we conducted a western blot analysis to evaluate if the heightened UPS activity was attributable to increased proteasome subunit levels. This involved analyzing the levels of PAS-{2, 3, 5, 7} in worms treated with FUdR under proteotoxic stress conditions induced either by the presence of bortezomib or upon silencing *pas-1*, *pbs-2*, *pbs-6*, and *rpn-9* proteasome components. Remarkably, we noted that FUdR treatment from young adult stage resulted in the downregulation of PAS-{2, 3, 5, 7} (Fig 1F). This finding mirrors the observation in germline-less worms, known for enhanced proteostasis and downregulation of 26S subunits [28]. For this reason, we checked the translation levels in the FUdR-treated nematodes by employing the surface sensing of translation (SUnSET) method which measures puromycin integration into new proteins. We identified a marked decrease in active translation upon FUdR treatment (S1E Fig), which is probably responsible for the observed decrease in proteasome subunit levels. This effect is likely tied to germline disruption, as similar reduction was evident in the germline-less, temperature-sensitive *glp-1(e2144)* mutant worms (S1E Fig). The reduction in protein synthesis observed in *glp-1(e2144)* mutants and FUdR-treated *C*. *elegans* might be attributed to the absence of a protein pool actively translated in the oocytes. To investigate whether this reduction also occurs in somatic tissues, we utilized a *C*. *elegans* strain expressing an endogenously mCherry-tagged 40S ribosomal subunit (RPS-6-mCherry). In this model, RNAi-mediated knockdown of the translation factors *iff-1* and *ifg-1* led to a decrease in RPS-6 signaling, reflecting diminished protein synthesis [42]. This decrease corresponded to reduced ribosome abundance, quantified across whole worms to monitor changes in the translation rate. To specifically assess the impact of FUdR on protein synthesis in somatic tissues, we monitored RPS-6-mCherry levels. Our results indicated that a 40-hour FUdR treatment starting from young adulthood affects protein synthesis, as evidenced by a decrease in mCherry signal across whole worms (S1F Fig). To exclude any contributions from the gonad, we focused our measurements on the head region. Here, a significant decrease in mCherry fluorescence was also observed following FUdR treatment (S1G Fig), confirming a reduction in protein synthesis within somatic tissues. Thus, despite the reduced synthesis of new proteasome subunits, FUdR-treated worms effectively elevated their proteasome activity.

### FUdR enhances proteasome function independent of germline proliferation and spermatogenesis

Given that FUdR treatment results in sterility in worms, we examined whether the observed effect was a consequence of FUdR-induced sterility. To this end, worms with RNAi-silenced *pbs-6* were subjected to varying concentrations of FUdR and other pyrimidine analogs at the young adult stage for 40 hours [43]. Our findings revealed that the UPS-supporting effect of FUdR was confined to concentrations that induced nematode sterility (min. 2 μM; S2A Fig), and a similar enhancement in the UPS performance was also observed with other pyrimidine analogs (S2A Fig). Former studies have underscored the essentiality of a unique proteasome lid subunit, RPN-6.1, in facilitating an upsurge in the UPS activity in germline lacking *glp-1* mutants [28]. Concurrently, sterile worms also necessitate the overexpression of a proteasome core α subunit, PBS-5, to foster proteostasis [9]. Because FUdR treatment catalyzes sterility in worms, we conjectured that RPN-6.1 and PBS-5 may be instrumental in the augmentation of proteostasis within FUdR-treated worms.

**Fig 2:**
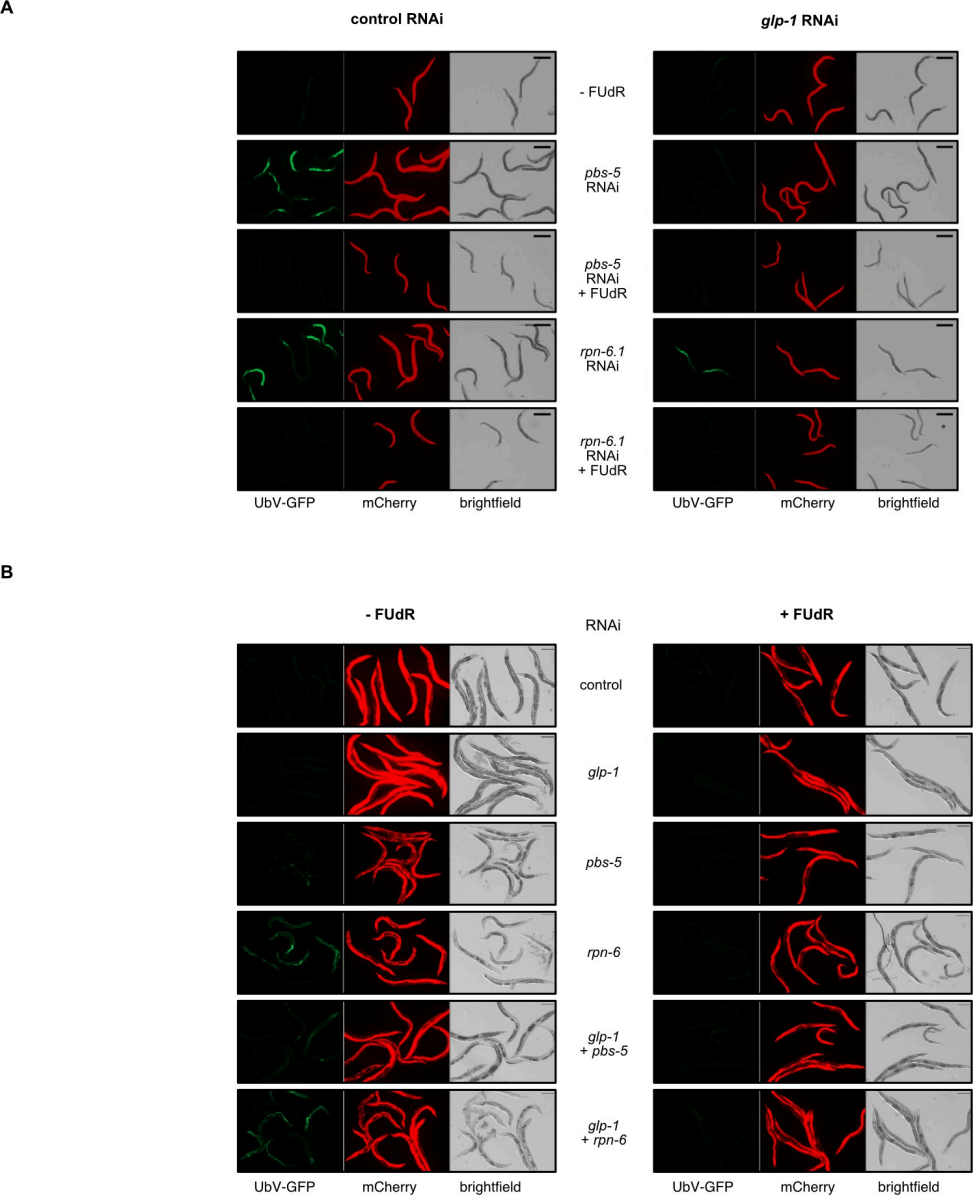
FUdR enhances proteasome function independent of germline proliferation. **(A)** FUdR impact on UbV-GFP turnover was examined in adult day 1 worms subjected to control, *pbs-5*, *rpn-6*.1, and double RNAi of *glp-1* with *pbs-5* or *rpn-6*.*1*, with or without FUdR treatment. *glp-1* silencing was initiated from the L1 stage, while double RNAi silencing of *glp-1* with *pbs-5* or *rpn-6*.*1*, along with FUdR treatment, was conducted from the young adult stage for 40 hours. Scale bar corresponds to 400μm. **(B)** UbV-GFP turnover assessed in adult day 3 worms with or without FUdR treatment under the following conditions: control RNAi, *pbs-5* RNAi, *rpn-6*.*1* RNAi, and double RNAi of *glp-1* with *pbs-5* or *rpn-6*.*1* (applied at the L4 stage). Scale bar corresponds to 400μm.

To validate this hypothesis, we conducted similar as described before *in vivo* assays in the reporter strain, examining UbV-GFP accumulation under conditions involving *glp-1* RNAi treatment, either alone or in conjunction with *pbs-5* or *rpn-6*.*1* RNAi, and with the variable inclusion of FUdR (Fig 2A). Despite *glp-1* RNAi ability to rescue the UbV-GFP accumulation phenotype under PBS-5 depletion conditions, it could not facilitate UbV-GFP turnover without RPN-6.1. In contrast, FUdR treatment succeeded in augmenting UPS activity by remedying UbV-GFP accumulation across all conditions. This suggests a divergence in the underlying mechanisms responsible for improved proteostasis between inhibition of germ cells proliferation by GLP-1 depletion and those rendered sterile by FUdR treatment. However, we observed a partial effect of *glp-1* RNAi, indicated by the presence of several eggs in the images (Fig 2A). Thus, it was essential to confirm that the impact of FUdR on proteostasis is independent of germline signaling. To this end, we conducted a comprehensive hatch size test in UbV-GFP worms after single and combined RNAi depletion of *glp-1*, *pbs-5*, and *rpn-6*.*1* from the L4 larval stage. The results showed that while the combined RNAi treatment effectively induced sterility (S2B and S2C Fig), the presence of FUdR supported UPS functionality and facilitated the degradation of its substrate (Fig 2B). Given that single GLP-1 depletion was not complete, and individuals continued to lay several eggs until day 2–3 (S2B Fig), we specifically focused on day-3 adults that no longer laid eggs to further reduce the impact of germline signaling and spermatogenesis on the FUdR effect. Our findings were consistent across these experiments, showing that FUdR still promoted UbV-GFP degradation under conditions of proteasome dysfunction (*pbs-5* and *rpn-6*.*1* RNAi depletion) and reduced germline proliferation (*glp-1* RNAi) in these older animals (Fig 2B). This suggests that the effect of FUdR on proteostasis operates through mechanisms distinct from those associated with the gonadal signaling pathway induced by GLP-1 depletion. In addition, despite prior assertions that FUdR-mediated resistance to proteotoxic stress is reliant on spermatogenesis [20], our findings suggest that the elevation of UPS activity facilitated by FUdR treatment is also not dependent on the spermatogenesis-associated factor FEM-1 or SPE-1 (S2D Fig).

### FUdR operates independently of conventional proteostasis regulators

To investigate whether the action of FUdR is dependent on a specific subunit of the proteasome, we analyzed the degradation of the UbV-GFP reporter in *C*. *elegans*. This was accomplished by immunoblotting lysates from bortezomib-treated worms, each with RNAi-mediated depletion of individual proteasome subunits. Intriguingly, our results indicated that the efficient turnover of UbV-GFP under FUdR treatment from the young adult stage does not rely on any specific proteasome subunit, as shown in (S3A Fig). This suggests that FUdR-induced mechanism of enhancing proteostasis may operate independently of the individual subunit composition of the proteasome.

**Fig 3:**
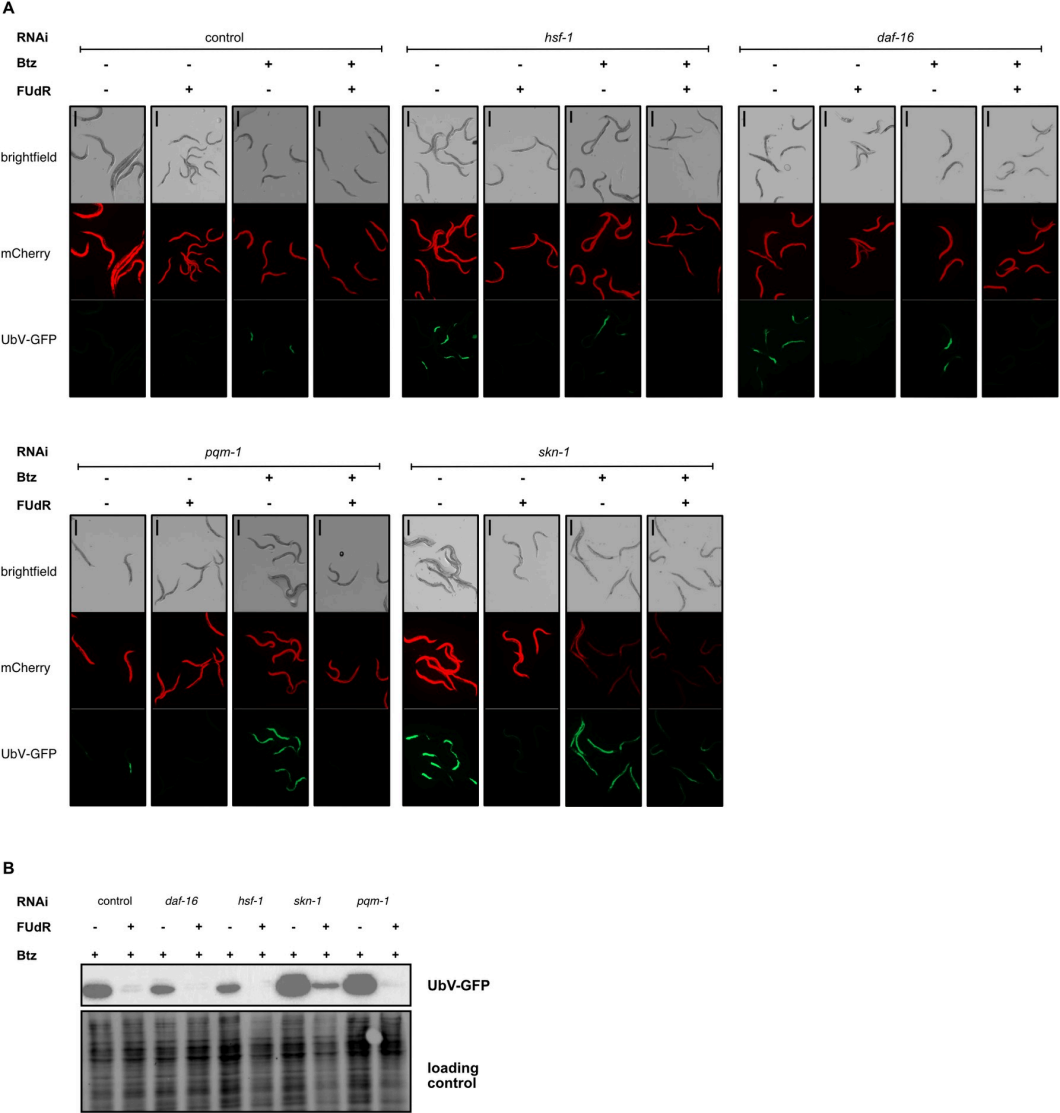
FUdR operates independently of conventional proteostasis regulators. **(A)** FUdR impact on UbV-GFP turnover in worms subjected to *hsf-1*, *daf-16*, *pqm-1*, and *skn-1* RNAi silencing in the absence or presence of bortezomib (Btz). In panels A and B, the scale bar corresponds to 400 μm. **(B)** Western blotting showing the effect of FUdR on UbV-GFP degradation in the worms subjected to *hsf-1*, *daf-16*, *pqm-1*, and *skn-1* RNAi silencing in the presence of bortezomib (Btz). Silencing of respective genes were carried out from L1 stage, whereas bortezomib and FUdR treatment was carried out from the young adult stage for 40 hours.

To discern the factors essential for UPS activity improvement via FUdR treatment, we induced silencing of *daf-16*, *hsf-1*, *pqm-1*, and *skn-1* factors recognized to regulate proteostasis and confer protection against proteotoxic stress [8,14,15,21,28], in the presence of bortezomib. FUdR treatment successfully corrected the UbV-GFP accumulation phenotype in cells devoid of HSF-1, DAF-16, and PQM-1 (Fig 3A and 3B). This observation aligns with previous research that revealed FUdR-mediated protection against proteotoxic stress independent of *hsf-1* and *daf-16* transcription factors [20]. Proteasomal dysfunction leads to the activation of the transcription factor SKN-1, essential for compensating proteasome function when proteasomal activity is compromised [29,30]. Interestingly, FUdR was able to restore UbV-GFP degradation even in the absence of SKN-1, achieved through RNAi-mediated depletion. Additionally, under conditions where SKN-1 was depleted and proteasomal activity was impaired by bortezomib, the addition of FUdR significantly enhanced substrate turnover (Fig 3A and 3B). To verify whether FUdR ability to bypass the absence of SKN-1 is associated with an increase in proteasome processivity, we measured this activity using lysates from wild-type worms treated with *skn-1* RNAi, with or without FUdR. Our data showed slight decreased chymotrypsin-like activity in the absence of SKN-1, consistent with previous studies [14]. Interestingly, the presence of FUdR did not increase it (S3B Fig), suggesting that FUdR activates an alternative UPS-stimulating or UPS-relieving pathway that functions independently of direct improvement of proteasome activity.

### FUdR prolongs lifespan and promotes cold survival under proteasome deficiencies

**Fig 4:**
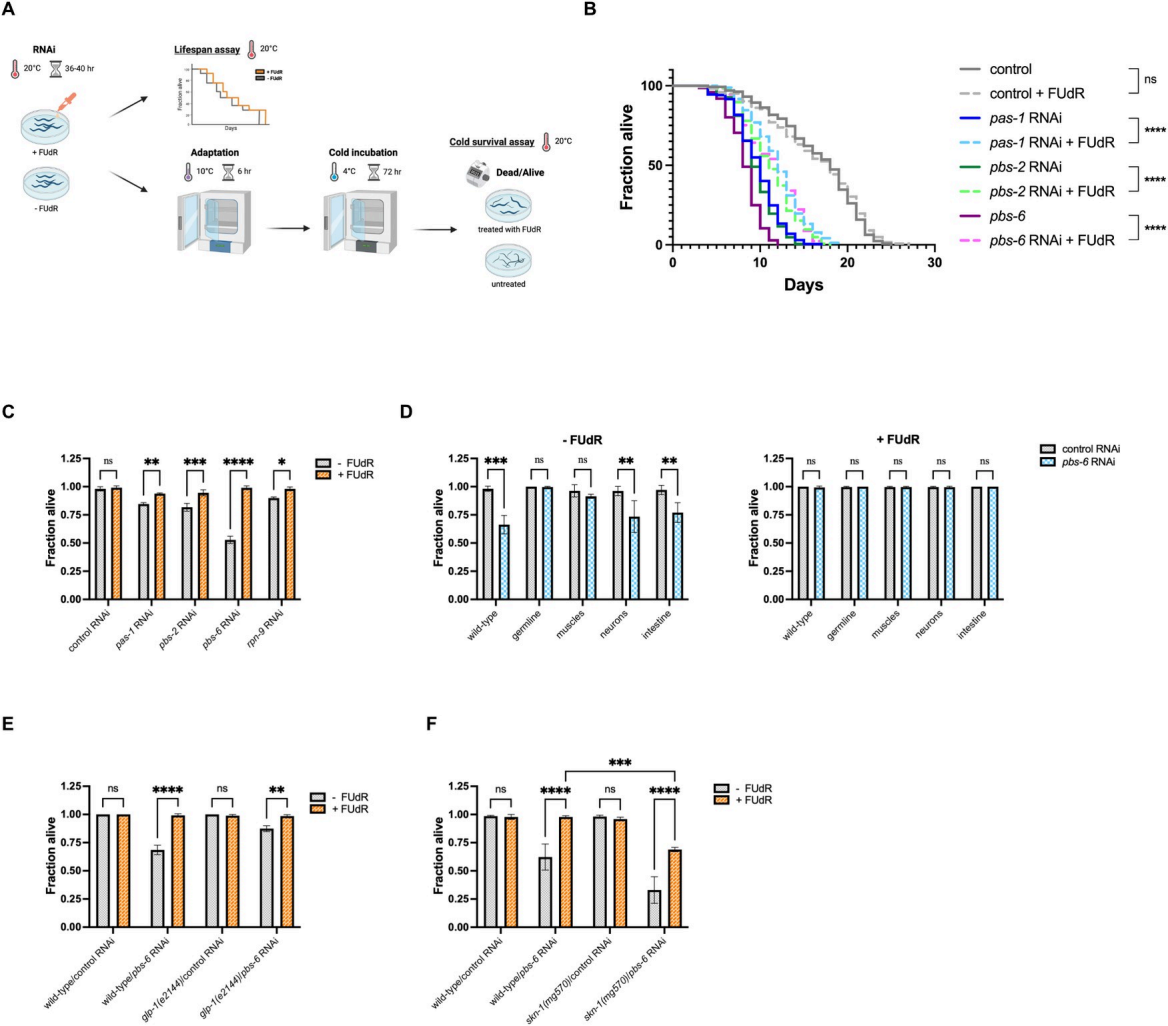
FUdR enhances lifespan and cold survival upon proteasome deficits. **(A)** A schematic representation of cold survival and lifespan assays. Created with BioRender.com **(B)** The lifespan of wild-type worms when exposed to control, *pas-1*, *pbs-2* or *pbs-6* RNAi in the presence or absence FUdR. S4 Table provides detailed lifespan data and associated statistics. **(C)** FUdR impact on the cold survival rate of wild-type worms during the knockdown of PAS-1, PBS-2, PBS-6, and RPN-9, considering both FUdR-treated and untreated conditions. Data was analyzed using two-way ANOVA and the significance levels obtained from the Šidák’s multiple comparisons test are indicated for the compared conditions (ns—not significant, *—*P* ≤ 0.05, **—*P* ≤ 0.01, ***—*P* ≤ 0.001, ****—*P* ≤ 0.0001). **(D)** FUdR impact on the cold survival of wild-type and *glp-1(e2144)* worms subjected to control and *pbs-6* RNAi. Data was analyzed using two-way ANOVA and the significance levels obtained from the Šidák’s multiple comparisons test are indicated for the compared conditions (ns—not significant, **—*P* ≤ 0.01, ****—*P* ≤ 0.0001). **(E)** FUdR impact on the cold survival of wild-type and *skn-1(mg570)* worms subjected to control and *pbs-6* RNAi. Data was analyzed using two-way ANOVA and the significance levels obtained from the Tukey’s multiple comparisons test are indicated for the compared conditions (ns—not significant, ***—*P* ≤ 0.001, ****—*P* ≤ 0.0001). In panels C-E, at least 90 animals were scored in three independent biological replicates. In panels B, C, D, E, and F, FUdR treatment was initiated at the young adult stage.

The successful induction of hibernation in *C*. *elegans* under cold conditions is contingent on a β subunit of the proteasome, PBS-6 [27]. Also, prior studies have underscored the indispensability of proteasome components for the longevity of worms [44]. In the light of our results that highlight FUdR’s capacity to amplify proteasome activity despite its deficits, we conducted cold-survival and lifespan assays (Fig 4A) on wild-type *C*. *elegans*, with simultaneous RNAi depletion of PAS-1, PBS-2, or PBS-6 proteasome subunits. Our data delineated that FUdR treatment notably increased the lifespan of worms subjected to proteasome disruption (Fig 4B). Additionally, we discovered that FUdR treatment from the young adult stage could effectively re-establish the survival of worms in cold survival assay, even amid proteotoxic stress induced by the depletion of PAS-1, PBS-2, PBS-6, and RPN-9 (Fig 4C). Notably, during cold survival assay, *C*. *elegans* requires a brief adaptation period at 10°C before exposure to cold incubation at 4°C. The absence of this adaptation period, followed by an abrupt cold incubation for 24 hours or more, has been proven lethal for the worms [45]. Hence, we conducted the cold survival assay with or without the adaptation period in the presence of FUdR, under proteasome malfunction caused by knockdown of PBS-6. The FUdR treatment significantly increased worm survival upon sudden cold shock (without an adaptation period), even under compromised proteasome function (S4A Fig).

To scrutinize the impact of tissue-specific proteostasis collapse on cold survival, we silenced *pbs-6* in wild-type strains and strains permitting tissue-specific RNAi depletion in muscles, intestines, germline, or neurons, again with or without FUdR. Our findings revealed that the knockdown of PBS-6 in neurons and intestines had the most pronounced impact on cold survival, which can be fully rescued by the FUdR treatment (Fig 4D).

Subsequently, we posited that FUdR-induced sterility plays a role in enhancing cold survival, a hypothesis supported by experiments involving both *glp-1(e2144)* sterile mutants and wild-type worms. The cold survival assay indicated enhanced survival in *glp-1(e2144)* mutants compared to wild-type worms when PBS-6 was depleted (Fig 4E). Moreover, this survival advantage of *glp-1(e2144)* worms was further potentiated in the presence of FUdR, suggesting that the mechanisms of cold tolerance induced by FUdR are likely distinct from the protective effects conferred by inhibition of germline proliferation.

As previously demonstrated, FUdR can restore UPS functionality amid proteasome dysfunction without reliance on the transcription factor SKN-1 (Fig 3A and 3B). To determine if this system remains effective under cold stress, we conducted a cold survival assay using both wild-type and *skn-1(mg570)* loss-of-function mutant, with or without RNAi silencing of *pbs-6* and FUdR treatment. While the *skn-1(mg570)* worms exhibited no notable decline in survival after cold incubation in the control RNAi condition, a significant reduction in survival was noted for *skn-1(mg570)* mutants when subjected to proteasome dysfunction by *pbs-6* silencing (Fig 4F). The observed decline was however rescued by FUdR treatment (Fig 4F). Collectively, our findings demonstrate that FUdR stimulates an alternative pathway that enhances the UPS, operating independently of both GLP-1 and SKN-1 (Figs 2A, 2B, 3A, 3B, and 4F).

### FUdR induces a detoxification pathway, which buffers UPS during proteasome inhibition

**Fig 5:**
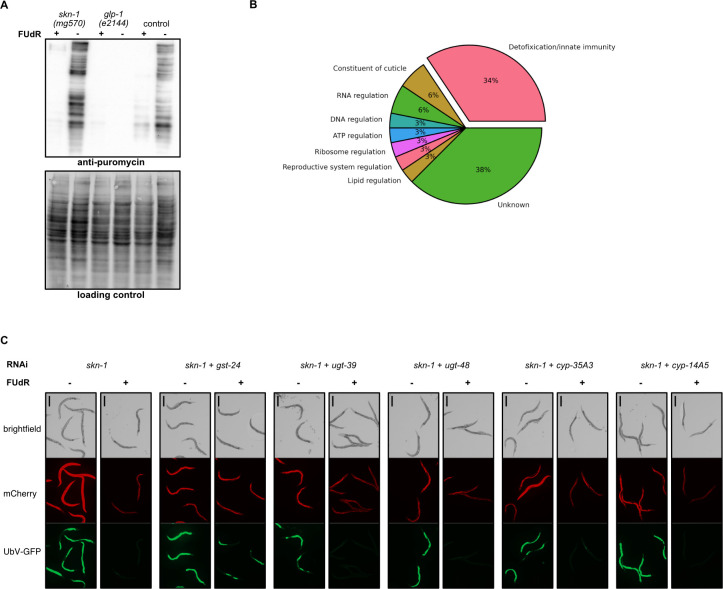
FUdR promotes detoxification pathway independent of *skn-1* and *glp-1*. **(A)** Western blot showing global translation activity in wild-type, *glp-1(e2144)*, and *skn-1(mg570)* worms, in the presence or absence of FUdR, as depicted by using anti-puromycin antibody. The No-Stain Protein Labeling Reagent was used to confirm equal protein loading. **(B)** Pie chart representing the functional categories of proteins from our proteomics study that exhibited a marked increase (fold change > 1.0), observed consistently in wild-type, *glp-1(e2144)*, and *skn-1(mg570)* worms specifically due to FUdR treatment. The significance of proteomic findings was confirmed through student’s t-tests, setting a *P*-value threshold < 0.05 while maintaining the false discovery rate under 0.01. Functional annotations were compiled by manual review. **(C)** FUdR impact on UbV-GFP turnover in worms subjected to *skn-1* RNAi silencing alone or in combination with *gst-24*, *ugt-39*, *ugt-48*, *cyp35A3*, and *cyp14A5* RNAi silencing, with or without FUdR. Scale bar corresponds to 400 μm. In panels A and C, FUdR treatment was carried out from the young adult stage for 40 hours.

Based on our findings from the SUnSET assay (S1E Fig), we speculated that the suppressive action of FUdR on translation could play a significant role in supporting UPS activity, given the well-established challenge that newly produced proteins pose to the proteostasis machinery [46,47,48,49]. However, our findings show that while both FUdR and the *glp-1(e2144)* mutation significantly reduce translation (Figs 5A and S1E), FUdR clearly activates UPS amplification mechanisms in germline-free *glp-1* mutants, as evidenced by its ability to override the RPN-6.1 requirement (Fig 2A and 2B). Similarly, in the *skn-1(mg570)* mutant, where we observed slightly higher protein synthesis compared to the control, FUdR almost completely blocked puromycin incorporation into elongating peptides (Fig 5A). Prior research has demonstrated that in response to global translation inhibition, ribosome availability facilitates selective translation, especially of stress response factors [48,49,50,51]. Given these insights, we hypothesized that the mechanism underlying FUdR action could be centered on the selective translation of the UPS buffering proteins, which should also occur in *glp-1* and *skn-1* mutants. To investigate this, we conducted a targeted proteomic analysis of adult wild-type, *glp-1(e2144)*, and *skn-1(mg570)* worms treated with 400 μM FUdR, aiming to identify proteins whose level is uniquely increased by FUdR. We identified and consistently quantified 5855 protein groups across all experimental conditions and three independent biological replicates. The analysis revealed that the FUdR treatment affected total levels of numerous proteins (717 up- and 821 down-regulated in wild-type, 416 up- and 442 down-regulated in *glp-1(e2144)*, 1132 up- and 1061 down-regulated in *skn-1(mg570)* worms) (S1 Table).

To uncover pathways activated solely by FUdR, we manually categorized functions of proteins up-regulated across all three strains upon FUdR treatment (S5A Fig and S2 Table). Our analysis revealed that over 33% of these proteins are implicated in detoxification and innate immunity pathways (Fig 5B), indicating that FUdR induces a detoxification response that is autonomous of both germline proliferation and SKN-1 activity. To eliminate the possibility that this induction is due to metabolites produced by bacterial metabolism of FUdR or bacterial infection, we examined UbV-GFP reporter degradation in worms fed with both live and killed HT115 *Escherichia coli*, in the presence or absence of bortezomib and FUdR. Our results demonstrated that FUdR effectively mitigated UbV-GFP accumulation under both bacterial conditions (S5B Fig).

A previous study has suggested that dopamine signaling induces the xenobiotic stress response and improves proteostasis [52]. To validate whether the neuronal signal is essential in improving the UPS activity upon FUdR induction, we measured the turnover of UbV-GFP reporter upon depleting the sensory neuron ciliary components CHE-12 and CHE-13 [53,54], with or without knockdown of SKN-1. Our findings indicate that individual silencing of either *che-12* or *che-13* exerts no discernible impact on UPS activity (S5C Fig). Simultaneous silencing of SKN-1 with either CHE-12 or CHE-13 reduced FUdR effectiveness in ameliorating UbV-GFP degradation, especially when targeting CHE-12 (S5C Fig). This suggests that neuronal signaling contributes to the regulation of proteostasis by FUdR under conditions of SKN-1 knockdown. To further elucidate our proteomic findings and clarify if detoxification proteins are involved in FUdR-mediated proteostasis enhancement, we focused on RNAi-mediated depletion of the most abundant detoxifying proteins—specifically GST-24, UGT-39, UGT-48, CYP-35A3, and CYP-14A5—that were uniquely up-regulated under FUdR treatment, while monitoring the turnover of the UBV-GFP reporter. Our data showed that GST-24 and CYP-14A5, when depleted individually, can affect UPS activity (S5D Fig). Notably, FUdR effectively reinstates proteasomal degradation in worms where SKN-1 is silenced alone or combined with UGT-39, UGT-48, CYP-35A3, or CYP-14A5 (Fig 5C). This restorative effect is absent when GST-24 is co-depleted with SKN-1 (Fig 5C), emphasizing the contribution of GST-24 to FUdR’s UPS enhancement following SKN-1 depletion. However, when GST-24 is depleted, it does not undermine the protective effect of FUdR on the worms resistance to low temperatures following PBS-6 depletion (S5E Fig). Due to the vast number of detoxifying genes in the genome, significant functional redundancy likely exists among these components. As such, this redundancy can make it challenging to observe the complete phenotype in any single RNAi experiment.

## Discussion

While FUdR is primarily known for inducing reproductive arrest, it also exerts secondary effects on various facets of *C*. *elegans* physiology, such as proteostasis and stress response mechanisms [16,19,20,21,22]. Our data indicated that FUdR augments the proteasome’s activities, akin to the effects observed in the sterile *glp-1(e2144)* mutant [28]. Although both FUdR and germline-deficient worms are known to elevate proteasome activity and stress resistance [13,22], FUdR appears to act independently of germline signaling. Enhanced proteostasis in *glp-1* mutants is contingent upon RPN-6.1, a lid subunit of the 26S proteasome [28], whereas the effect of FUdR is not. Moreover, these mechanisms further diverge: while *glp-1* mutants are dependent on *daf-16* for both lifespan extension and elevated proteasome activity [28], the enhancement of UPS activity in FUdR-treated worms is not attributable to the *daf-16* transcription factor. Proteasomal dysfunction activates the transcription factor SKN-1, which is essential for maintaining proteasome function under compromised conditions [29,30]. Yet, we observed that FUdR restored the degradation of the ubiquitin fusion reporter even in the absence of SKN-1. Furthermore, when SKN-1 was depleted and the proteasome was impaired by bortezomib, FUdR was able to partially induce substrate turnover. Interestingly, FUdR did not increase proteasome activity under SKN-1 depletion conditions, suggesting that FUdR activates an alternative pathway that stimulates or relieves the UPS, independent of directly enhancing proteasome activity. Although SKN-1 generally activates proteasome subunits [14,55], FUdR does not rely on specific 26S proteasome subunits or autophagy, aggrephagy, or chaperone-mediated autophagy for its effect in removing UPS model substrates. The effect of FUdR could potentially depend on deubiquitinating enzymes (DUBs) in the proteasomal degradation of ubiquitinated substrates, as enhancement of proteasomal degradation might be achieved by their inhibition [56]. Alternatively, down-regulation of proteasome-associated DUBs, such as UBH-4, could increase proteasome activity in *C*. *elegans* [34]. Since FUdR administration decreases translation, including that of proteasome subunits, while improving proteasome activity, it is plausible that this effect is mediated by a mechanism based on a drop in UBH-4 levels.

Our findings demonstrated that FUdR treatment increases levels of various xenobiotic detoxifying proteins, including those involved in the innate immune response, independently of SKN-1 and GLP-1. Previous research has shown that the turnover of the unstable UbV-GFP reporter and cellular UPS activity are influenced by xenobiotic detoxifying enzymes, and that sensory neuron signaling partially modulates proteostasis by promoting xenobiotic metabolism [52]. We observed that FUdR enables efficient UPS substrate turnover under SKN-1 dysfunction, presumably through this mechanism. Specifically, the normal structure and function of sensory cilia and the GST-24 protein, previously linked to stress resistance in *C*. *elegans* [48], play a significant role in this process. Furthermore, given that FUdR induces DNA breaks, activating damage repair pathways [24], and that DNA breaks in the germline improve UPS activity through an innate immune response [57], it is plausible that FUdR triggers this detoxification pathway in response to DNA damage.

Building on research regarding the physiological effects of FUdR in animals with a dysfunctional proteasome, we focused on its impact on lifespan and cold tolerance. Early studies indicated that hibernation in *C*. *elegans* at low temperatures (4°C) depends on the β subunit of the proteasome, PBS-6 [27]. Furthermore, proteasome components are crucial for worm longevity [33]. Therefore, we performed survival assays at 4°C and assessed animal longevity at 20°C with simultaneous RNAi depletion of proteasome subunits. Our data showed that FUdR treatment significantly prolonged the lifespan of worms with proteasome disruption and effectively restored survival at low temperatures, even under proteotoxic stress induced by depletion of PAS-1, PBS-2, PBS-6, and RPN-9. We hypothesized that FUdR’s inhibition of germ cell proliferation might enhance survival at low temperatures. However, the survival of *glp-1(e2144)* worms with depleted PBS-6 was further enhanced by FUdR, suggesting that the cold tolerance mechanisms induced by FUdR are distinct from those associated with germ cell proliferation inhibition. Recent findings have shown that at moderately low temperatures (15°C), the germline promotes longevity. Proliferating germ cells induce cystathionine β-synthase (*cbs-1*) in somatic tissues, which extends longevity at this temperature. Notably, FUdR treatment reduces high *cbs-1* expression in somatic tissues at 15°C, thereby shortening longevity [58]. However, in our experimental setup, we did not observe a decrease in CBS-1 protein levels after FUdR treatment under any conditions (N2, *glp-1* and *skn-1* mutants). This maintenance of CBS-1 levels may allow FUdR-treated animals to adapt and extend their longevity at 4°C. Further investigation is warranted to determine whether, in the context of proteasome dysfunction and SKN-1 mutation, CBL-1 is also involved in the protective mechanism triggered by FUdR. Interestingly, GST-24, a protein from the detoxification pathway identified as important for FUdR’s UPS buffering effect upon SKN-1 depletion, is not essential for FUdR protective effect on worm resistance to 4°C following PBS-6 depletion. This suggests significant functional redundancy among detoxifying genes, making it challenging to observe the complete phenotype in any single RNAi experiment.

Parallelly, we observed that treatment with FUdR enhances the ability of *C*. *elegans* to withstand cold stress at 4°C, even bypassing the usual need for a preparatory acclimatization phase at 10°C. This raises the possibility that FUdR treatment mimics or induces adaptive processes normally activated during this acclimatization period. Previous research has shown that moderate cold exposure at 10°C triggers shifts in lipid metabolism, which are likely crucial for enhanced cold resistance [59,60]. Interestingly, lipid metabolism in *C*. *elegans* is deeply interwoven with innate immune responses; specific fatty acids and their synthesizing enzymes are essential for immune gene expression and pathogen resistance [61]. Given that FUdR pre-treatment activates detoxification pathways in the worms prior to exposure to severe cold, it is conceivable that such pathways and lipid metabolism may interact to confer cold resilience and proteostasis disruption.

It is noteworthy that the beneficial effects of FUdR in promoting worms survival and protein turnover are not dependent on evolutionarily preserved transcriptional regulators DAF-16 and PQM-1, recognized for their cooperative role in enhancing resilience to cold conditions through the upregulation of FTN-1/ferritin [62]. This delineates a distinct mechanistic route through which FUdR modulates cellular homeostasis and also raises intriguing questions about the potential cross-talk between various stress response pathways and how they might be selectively activated or bypassed.

In conclusion, our study provides a comprehensive analysis of the diverse functional roles of FUdR in *C*. *elegans*, emphasizing its modulation of the UPS efficiency, detoxification pathways, and cold resilience mechanisms. Although there are marked differences in the architecture of immune systems between nematodes and higher organisms, several innate immunity and detoxification mechanisms are evolutionarily preserved, suggesting potential translational relevance [63]. These findings thus warrant further exploration of the influence of FUdR on proteasomal activity, particularly in the context of its co-administration with agents like bortezomib in oncological patients [64,65]. Additionally, it would be valuable to study whether FUdR or related molecules have the potential to enhance cellular resilience in mammals subjected to environmental stressors, including temperature fluctuations. Such research could extend our grasp of the adaptability-promoting properties of these compounds across different species.

## Materials and methods

### Worm maintenance

*C*. *elegans* were cultured on nematode growth medium (NGM) plates seeded with OP50 or HT115 *E*. *coli* strains. These culture conditions were maintained at various temperatures, namely 16°C, 20°C, or 25°C, depending on both the worm strain and the specific requirements of the experimental protocol, following standard *C*. *elegans* culture techniques [66]. Dead bacterial food sources were prepared by exposing bacterial cultures to paraformaldehyde [67]. Worm strains utilized for different experimental setups are cataloged in Table 1. Unless stated otherwise, NGM plates were supplemented with 400 μM of floxuridine (FUdR; Cat: F0503; Sigma Aldrich). In some instances, other pyrimidine analogs were employed, including 5-fluorocytosine (FC; Cat: 543020; Sigma Aldrich), 5-fluorouracil (FU; Cat: F6627; Sigma Aldrich), and 5-fluoro-2′-deoxycytidine (FCdR; Cat: F5307; Sigma Aldrich), as well as proteasome inhibitor bortezomib (Cat: 504314; Sigma Aldrich), each at a concentration of 10 μM.

**Table 1:** *C*. *elegans* strains used in this study.

Strain	Source	Purpose
*C*.* elegans*: Bristol (N2)	CGC^1^	Wild-type; control worms
*skn-1(mg570)*	CGC	*skn-1* mutant
*glp-1(e2144)*	CGC	*glp-1* mutant
hhls72*[unc-119(+); sur-5*::*mCherry]*, hhls64*[unc-119(+); sur-5*::*UbiV-GFP]*	[33]	UPS activity reporter
*rde-1(mkc36);* mkcSi13	[68]	Germline-specific RNAi
*rde-1(ne219)*; kzls20	[69]	Body wall muscle-specific RNAi
*rde-1(ne219);* kbls7	[70]	Intestine-specific RNAi
TU3311 *[uls60 (unc-119p*::*YFP + unc-119p*::*sid-1)]*	[71]	Neuron-specific RNAi
vha-6p::UIM2-ZsProSensor	[34]	Polyubiquitin protein reporter
*rps-6*(*rns6*[*rps-6*::mCherry]) I	[42]	Translation reporter

^1^ Caenorhabditis Genetics Center

### RNA interference

Gene silencing was carried out by the standard RNAi feeding method using clones from the Ahringer *C*. *elegans* RNAi library [72]. NGM plates supplemented with 1 mM IPTG (Cat: IPT001; BioShop) and 25 μg/μl carbenicillin (Art. No. 6344.2; Carl Roth Gmbh & Co.) were seeded with bacteria expressing double-stranded RNA from L4440 plasmid; *E*. *coli* HT115 (DE3) bacteria containing empty L4440 plasmid were used as control. The designated worm stages for specific RNAi treatments are detailed in S3 Table.

### Lifespan assay

Synchronized young adult worms were silenced for *pbs-6*, *pas-1*, *pbs-2*, or control RNAi (empty vector) with or without FUdR throughout the lifespan. Subsequently, lifespan measurements were carried out at a constant temperature of 20°C. Approximately 30 nematodes were maintained on each 6 cm diameter agar plate for the duration of the lifespan assay. Daily evaluations were performed to monitor the worms for movement and pharyngeal pumping as vitality indicators. Worms were deemed to have reached the end of their lifespan if they failed to display these physiological activities. Any animals manifesting bagging phenotypes or found to have crawled off the agar surface were censored. Data was analyzed using the Log-Rank (Mantel-Cox) test in the GraphPad Prism 9 software. Statistical data of individual lifespan experiments is presented in S4 Table. The experiments were not randomized. No statistical methods were used to predetermine the sample size. The investigators were blinded to allocation during experiments.

### Brood size assay

Age-synchronized L4 hermaphrodites of the hhIs72[*unc-119*(+); *sur-5*::mCherry]; hhIs64[*unc-119*(+); *sur-5*::UbV-GFP] [31] strain were selected and subjected to RNAi treatments targeting *glp-1*, *pbs-5*, *rpn-6*.*1*, *glp-1+pbs-5*, *glp-1+rpn-6*.*1*, or a control RNAi (empty vector), with or without FUdR treatment. For the assay, two worms were placed on each 35mm NGM plate. Brood size measurements were conducted at a constant temperature of 20°C. Worms were transferred to fresh NGM plates daily, from adulthood day 0 to adulthood day 3, marking the end of the egg-laying period. The number of eggs laid, and larvae hatched were manually counted each day throughout the reproductive stage. For each biological repeat, eggs laid, and larvae hatched from 10 worms were counted. Data are presented as the total number of eggs laid and larvae hatched per plate, aggregated across three independent biological repeats.

### Cold survival assay

To assess the impact of specific gene knockdown and FUdR treatment on cold tolerance, approximately 30 young adult worms *C*. *elegans* were subjected to gene silencing. The gene silencing was performed for 40 hours at a controlled temperature of 20°C, in the presence or absence of FUdR, according to protocols detailed in the study by Habacher and colleagues [27].

#### Adaptation and exposure to cold conditions

Following the gene silencing phase, the nematodes were acclimatized to a moderately cold environment at 10°C for 6 hr. Subsequently, the worms were exposed to a colder temperature setting at 4°C and maintained at this condition for 72 hr.

#### Recovery and scoring

After the 3-day cold exposure, the worms were allowed to recover at 20°C, lasting between 3 and 4 hr. Following the recovery phase, an assessment was carried out to distinguish between live and dead specimens.

#### Cold incubation treatment

As a control, cold incubation was administered by directly transferring the worms from a 20°C environment to a 4°C setting for 72 hours without prior acclimatization.

#### UbV-GFP substrate turnover assay

To explore the turnover dynamics of the proteasome substrate (UbV-GFP) under gene knockdown and pharmacological treatments, we utilized L4 larval or young adult stage hermaphrodites of the strain hhIs72[*unc-119*(+); *sur-5*::mCherry]; hhIs64[*unc-119*(+); *sur-5*::UbV-GFP] [31]. Target gene silencing was carried out for approximately 40 hours at a consistent temperature of 20°C. During this period, worms were treated with varying concentrations of FUdR, FU, FC, FCdR, and 100 nM bortezomib. Post-treatment, nematodes were subjected to fluorescence imaging using an Axio Zoom.V16 microscope equipped with an Axiocam 705 monochrome CMOS camera (Carl Zeiss). Images were captured in brightfield, green, and red channels. Data processing was subsequently performed using AxioVision 4.7 analysis software (Carl Zeiss).

### Monitoring the level of polyubiquitinated proteins

Age-synchronized L4 hermaphrodites of the *vha-6*p::UIM2-ZsProSensor strain [34] were cultured in the presence or absence of FUdR and bortezomib for 40 hours at 20°C. Fluorescence imaging was then performed using an Axio Zoom.V16 microscope equipped with an Axiocam 705 monochrome CMOS camera (Carl Zeiss). Images were captured across brightfield, green, and red channels. The normalized fluorescence intensity of whole worms was quantified using ImageJ software. Data are presented from 19 worms across three independent biological repeats.

### Proteasome activity measurement in worms

Approximately 7000 young adult stage worms were silenced for the respective genes at a concentration of 400 μM for 40 hours at 20°C. Next, worms were harvested and lysed in 50 mM Tris-HCl, 250 mM sucrose, 5 mM MgCl_2_, 0.5 mM EDTA, 2 mM ATP, and 1 mM dithiothreitol at pH 7.5. Bortezomib was introduced to the lysate as a control at a concentration of 20 nM. The caspase-like, chymotrypsin-like, and trypsin-like activities of the proteasome were subsequently measured following the methods described [28]. Proteasome activity was also measured using 5 nM of recombinant human 26S proteasome (Cat: E-365; R&D Systems).

### Proteasome activity measurement in cells

HeLa Flp-In T-Rex cell line (a kind gift from Roman Szczęsny) was cultured in Dulbecco’s Modified Eagle’s Medium (Cat: 6429; Sigma Aldrich) supplemented with 10% fetal bovine serum (Cat: F9965; Sigma Aldrich) and 1% Antibiotic-Antimycotic (Cat: 15240096; Gibco) at 37°C with 5% CO_2_ in a humidified incubator. HeLa cells were seeded in black 96-well tissue culture plates (Cat: 655090; Greiner) at a density of 20,000 cells in a total volume of 100 μl per well. The next day, the cells were subjected to a 6-hour treatment with FUdR at 2.5 μg/ml concentration in the growing media. Where indicated, the cells were also treated with 10 nM bortezomib (Cat: 504314; Merck). The control cells received dimethyl sulfoxide. Using the Proteasome 20S Activity Assay Kit (Cat: MAK172; Sigma Aldrich), 10.75 μl of LLVY-R110 Substrate was mixed with 4300 μl of the Assay Solution to create the Proteasome Assay Loading Solution, according to the manufacturer’s guidelines. Subsequently, 100 μl of this prepared solution was accurately pipetted into each assay plate well. The plate was then incubated at 37°C for 2 hr. After the incubation period, the fluorescence intensity (λ_ex_ = 490 nm, λ_em_ = 525 nm) was gauged using the TECAN Infinite 200 Pro plate reader equipped with the Magellan Pro software to ascertain proteasome activity.

### Monitoring global translation

#### Surface sensing of translation assay

The surface sensing of translation (SUnSET) assay was employed to evaluate protein synthesis rates, following the delineated methodology [73,74]. A minimum of 7,000 worms were incubated in 4 mL of S-complete liquid media supplemented with 750 μL of 10X *E*. *coli* food, engineered to express double-stranded RNA against targeted genes, and 0.5 mg/mL puromycin (Cat: 58-58-2, ant-pr-1; Invivogen). The incubation occurred at 20°C with a consistent shaking speed of 200 rpm for 5 hr. The worms were then harvested, washed three times in M9 buffer, and subsequently lysed in a lysis buffer (50 mM KCl, 10 mM Tris-HCl pH 8.2, 2.5 mM MgCl_2_, 0.07% NP-40, 0.7% Tween-20, 0.1% gelatin), supplemented with protease inhibitor (Cat: 04693159001; Roche). Protein concentration was determined using the Pierce Rapid Gold BCA Protein Assay Kit (Cat: A53225; Thermo Fisher Scientific) and western blotting.

#### Fluorescence microscopy of translation reporter

Translation was monitored in the *rps-6*(*rns6*[*rps-6*::mCherry]) reporter strain [42] by observing mCherry fluorescence after culturing young adult worms in the presence or absence of FUdR for 40 hours at 20°C. Fluorescence imaging was performed using an Axio Zoom.V16 microscope equipped with an Axiocam 705 monochrome CMOS camera (Carl Zeiss). Images were captured across brightfield and red channels. The normalized fluorescence intensity of whole worms and the head region were quantified using ImageJ software. Data are presented from 30 worms across three independent biological repeats.

### Western blotting

Proteins were separated by electrophoresis on 12% acrylamide gels using a running buffer containing 25 mM Tris, 190 mM glycine, and 0.1% SDS. Electrophoresis was conducted at a constant voltage of 150 V. Subsequently, protein samples were transferred to PVDF membranes through a wet transfer method at 100 V for one hour. The transfer buffer contained 25 mM Tris, 190 mM glycine, and 10% methanol, with a pH of 8.3. For protein visualization, the membranes were treated with Invitrogen No-Stain Protein Labeling Reagent (Cat: A44717; Thermo Scientific) according to the manufacturer’s guidelines. Following this, the membranes were blocked in a solution of 5% skimmed milk dissolved in TBST buffer (50 mM Tris, 150 mM NaCl, 0.1% Tween 20, pH 7.5) for 45 min at room temperature. Primary antibody incubation was performed overnight at 4°C using either anti-GFP antibody (Cat: GF208R; Thermo Fisher Scientific), anti-PAS-7 antibody (Cat: CePAS7; Developmental Studies Hybridoma Bank (DSHB)), anti-ubiquitin antibody (Cat:3936; Cell Signaling Technology), anti-proteasome 20S alpha 1+2+3+5+6+7 antibody (Cat: ab22674; Abcam) or anti-puromycin (Cat: MABE343; Merck), both prepared in 5% skimmed milk in TBST buffer. Post-incubation, membranes were washed three times with TBST for 10 min each and incubated with the appropriate secondary antibodies, prepared in the blocking solution, for 45 min at room temperature. Membranes were subsequently developed and visualized using a ChemiDoc Imaging System from Bio-Rad.

### Proteomics analysis

*C*. *elegans* were extracted using the Sample Preparation by Easy Extraction and Digestion (SPEED) protocol [75]. In brief, *C*. *elegans* were solubilized in concentrated Trifluoroacetic Acid (TFA; Cat: T6508; Sigma Aldrich) (cell pellet/TFA 1:2–1:4 (v/v)) and incubated for 2–10 min at room temperature. Next, samples were neutralized with 2 M Tris-Base buffer using 10x volume of TFA and further incubated at 95°C for 5 min after adding Tris(2-carboxyethyl)phosphine (final concentration 10 mM) and 2-chloroacetamide (final concentration 40 mM). Turbidity measurements determined protein concentrations at 360 nm, adjusted to the same concentration using a sample dilution buffer (2M TrisBase/TFA 10:1 (v/v)), and then diluted 1:4–1:5 with water. Digestion was carried out overnight at 37°C using trypsin at a protein/enzyme ratio of 100:1. TFA was added to a final concentration of 2% to stop digestion. The resulting peptides were labeled using an on-column TMT labeling protocol [76]. TMT-labeled samples were compiled into a single TMT sample and concentrated. Peptides in the compiled sample were fractionated (8 fractions) using the Pierce High pH Reversed-Phase Peptide Fractionation Kit (Cat: 84868; Thermo Fisher Scientific). Prior to liquid chromatography–mass spectrometry (LC-MS) measurement, the peptide fractions were reconstituted in 0.1% TFA, 2% acetonitrile in water. Chromatographic separation was performed on an Easy-Spray Acclaim PepMap column 50 cm long × 75 μm inner diameter (Cat: PN ES903; Thermo Fisher Scientific) at 55°C by applying 120 min acetonitrile gradients in 0.1% aqueous formic acid at a flow rate of 300 nl/min. An UltiMate 3000 nano-LC system was coupled to a Q Exactive HF-X mass spectrometer via an easy-spray source (all Thermo Fisher Scientific). The Q Exactive HF-X was operated in TMT mode with survey scans acquired at a resolution of 60,000 at m/z 200. Up to 15 of the most abundant isotope patterns with charges 2–5 from the survey scan were selected with an isolation window of 0.7 m/z and fragmented by higher-energy collision dissociation (HCD) with normalized collision energies of 32, while the dynamic exclusion was set to 35 s. The maximum ion injection times for the survey and tandem mass spectrometry (MS/MS) scans (acquired with a resolution of 45,000 at m/z 200) were 50 and 96 ms, respectively. The ion target value for MS was set to 3e6 and for MS/MS to 1e5, and the minimum AGC target was set to 1e3.

The data were processed with MaxQuant v. 1.6.17.0 [77], and the peptides were identified from the MS/MS spectra searched against Uniprot *C*. *elegans* reference proteome (UP000001940) using the built-in Andromeda search engine. Raw files from the liquid chromatography with tandem mass spectrometry (LC-MS/MS) measurements of 8 tryptic peptide fractions were analyzed together. Reporter ion MS2-based quantification was applied with reporter mass tolerance = 0.003 Da and min. reporter PIF = 0.75. Cysteine carbamidomethylation was set as a fixed modification, and methionine oxidation, glutamine/asparagine deamination, and protein N-terminal acetylation were set as variable modifications. For *in silico* digests of the reference proteome, cleavages of arginine or lysine followed by any amino acid were allowed (trypsin/P), and up to two missed cleavages were allowed. The false discovery rate (FDR) was set to 0.01 for peptides, proteins, and sites. A match between runs was enabled. Other parameters were used as pre-set in the software. Unique and razor peptides were used for quantification, enabling protein grouping (razor peptides are the peptides uniquely assigned to protein groups and not to individual proteins). Reporter intensity corrected values for protein groups were loaded into Perseus v. 1.6.10.0. [78]. Standard filtering steps were applied to clean up the dataset: reverse (matched to decoy database), only identified by site, and potential contaminant (from a list of commonly occurring contaminants included in MaxQuant) protein groups were removed. Reporter intensity corrected values were log2 transformed, and protein groups with all values were kept. Reporter intensity values were then normalized by median subtraction within TMT channels. Student’s t-tests (permutation-based FDR = 0.001, S0 = 0.1) were performed on the dataset to return protein groups, whose levels were statistically significantly changed between the sample groups investigated. The results of proteomics analysis, indicating changes in protein abundance in wild-type (N2), *glp-1(e2144)*, and *skn-1(mg570)* worms with or without FUdR treatment, are presented in S1 Table. Proteins up-regulated in wild-type (N2), *glp-1(e2144)*, and *skn-1(mg570*) upon FUdR treatment, along with their manually categorized functions, are detailed in S2 Table.

## Supporting information

S1 Table Results of proteomics analysis showing changes in protein abundance in wild-type, *glp-1(e2144)*, and *skn-1(mg570)* worms in the presence or absence of FUdR. (XLSX)

S2 Table Proteins up-regulated in wild-type, *glp-1(e2144)*, and *skn-1(mg570)* upon FUdR treatment with manually categorized functions. (PDF)

S3 Table The designated worm stages for specific RNAi treatments. (PDF)

S4 Table Statistical data of individual lifespan experiments. (XLSX)

S1 Fig FUdR buffers UPS activity under proteasome-compromised conditions. **(A)** Western blot showing the impact of FUdR on the accumulation of poly-ubiquitinated proteins in the presence of control RNAi or *pbs-6* RNAi using anti-ubiquitin antibody. The No-Stain Protein Labeling Reagent was used to confirm equal protein loading. Silencing of *pbs-6* and FUdR treatment was carried out from the young adult stage for 40 hours. **(B)** FUdR effect on the activity of purified human 26S proteasome as measured by chymotrypsin-like activity in the presence and absence of FUdR. Bortezomib (Btz) served as the negative control. Proteasome activity is represented as slopes obtained from kinetic measurements. The experiments were conducted thrice as separate biological replicates. **(C)** FUdR effect on chymotrypsin-like proteasome activity in HeLa cells. Cells were treated with final concentrations of 0.4 and 2 μM of FUdR and 10 nM bortezomib (Btz) as control for 6 hr. The assay was conducted by incubating the cells with 100 μl Proteasome Assay Loading Solution for 2 h as described in the methods. Results from three technical replicates were corrected for background by subtracting the fluorescence of the medium without cells and further normalized to dimethyl sulfoxide control. The graph shows the average values obtained from either two or four biological replicates for experiments that involve FUdR or Btz. **(D)** Western blot showing degradation of UbV-GFP in control, *atg-1* RNAi (applied at either L1 or young adult stages), and *lgg-1* RNAi (applied at the young adult stage) worms co-treated with bortezomib (Btz) in the presence or absence of FUdR, as depicted by using anti-GFP antibody. The No-Stain Protein Labeling Reagent was used to confirm equal protein loading. **(E)** Western blot showing global translation activity in wild-type and *glp-1(e2144)* worms, in the presence or absence of FUdR, as depicted by using the anti-puromycin antibody. The No-Stain Protein Labeling Reagent was used to confirm equal protein loading. (**F**) The level of RPS-6-mCherry was measured in adult nematodes across the entire body, with and without FUdR treatment (applied at the young adult stage for 40 hours). The scale bar corresponds to 400 μm. A graph is provided showing the normalized fluorescence intensity of the mCherry signal in the presence or absence of FUdR. n = 30 from three biological replicates, and significance levels (****- P<0.0001) were determined using Mann-Whitney test. (**G**) The level of RPS-6-mCherry was measured in the head region of adult nematodes, with or without FUdR treatment (applied at the young adult stage for 40 hours). The scale bar corresponds to 200 μm. A graph is provided showing the normalized fluorescence intensity of the mCherry signal. n = 30 from three biological replicates, and significance levels (****- P<0.0001) were determined using Mann-Whitney test.
(TIFF)

S2 Fig FUdR enhances UPS activity independently of germline proliferation and spermatogenesis. **(A)** The effect of various concentrations of FUdR and pyrimidine analogs, including 5-fluorouracil (FU), 5-fluorocytosine (FC), and 5-fluorodeoxycytidine (FCdR), on UbV-GFP turnover was assessed. The scale bar corresponds to 400 μm. FUdR treatment was applied at the young adult stage for 40 hours. **(B)** The graph shows the total number of eggs laid by UbV-GFP-expressing animals (2 animals per plate) in the presence or absence of FUdR, subjected to control, *pbs-5*, *rpn-6*.*1*, and double RNAi of (*glp-1 + pbs-5*) and (*glp-1 + rpn-6*.*1*) applied at the L4 stage. Data represent n = 30 from three independent biological repeats. Data was analyzed using two-way ANOVA and the significance levels obtained from the Šidák’s multiple comparisons test are indicated for the compared conditions (ns—not significant, *- P<0.05, **- P<0.01, ***-P<0.001, ****-P<0.0001). **(C)** The total number of larvae hatched by UbV-GFP expressing animals (2 animals per plate) was assessed in the presence or absence of FUdR, subjected to control, *pbs-5*, *rpn-6*.*1* and double RNAi of (*glp-1 + pbs-5*) and (*glp-1 + rpn-6*.*1*) applied at the L4 stage. In panels B and C, FUdR treatment was initiated at the L4 stage. Data represent n = 30 from three independent biological repeats. Data was analyzed using two-way ANOVA and the significance levels obtained from the Šidák’s multiple comparisons test are indicated for the compared conditions (ns—not significant, ****-P<0.0001). **(D)** Western blot showing degradation of UbV-GFP upon RNAi depletion of SPE-1 and FEM-1, with or without bortezomib (Btz) or FUdR, detected using an anti-GFP antibody. Equal protein loading was confirmed with the No-Stain Protein Labeling Reagent. FUdR treatment was applied from the young adult stage for 40 hours.
(TIFF)

S3 Fig FUdR promotes UbV-GFP turnover independently of proteasome subunit composition. **(A)** Western blot showing the impact of FUdR on UbV-GFP reporter turnover upon depletion of various 26S subunits in control worms treated with bortezomib (Btz), detected using an anti-GFP antibody. The No-Stain Protein Labeling Reagent confirmed equal protein loading. Bortezomib and FUdR treatments were carried out from the young adult stage for 40 hours. **(B)** FUdR effect on proteasome activity, measured by chymotrypsin-like activity in wild-type worms subjected to control RNAi and *skn-1* RNAi, with or without FUdR treatment (applied at the young adult stage for 40 hours). Proteasome activity is represented as slopes obtained from kinetic measurements. The experiments were conducted thrice as separate biological replicates, and significance levels (ns—not significant) were determined using an unpaired t-test with Welch’s correction.
(TIFF)

S4 Fig FUdR improves cold survival without adaptation period. **(A)** The impact of FUdR on the cold survival of wild-type and *pbs-6* knockdown worms, with or without a cold adaptation period. Data was analyzed using two-way ANOVA and the significance levels obtained from the Šidák’s multiple comparisons test are indicated for the compared conditions (ns—not significant, ****—*P* ≤ 0.0001).
(TIFF)

S5 Fig FUdR activates a detoxification pathway. **(A)** Venn diagram showing the abundance of proteins up-regulated exclusively in the presence of FUdR in wild-type, *glp-1(e2144)* and *skn-1 (mg570)* worms. **(B)** Western blot showing the impact of bacterial viability on the UbV-GFP reporter turnover in the presence of bortezomib (Btz) and FUdR, as depicted by using anti-GFP antibody. The No-Stain Protein Labeling Reagent was used to confirm equal protein loading. **(C)** Impact of FUdR and RNAi knockdown of neuronal ciliary components *che-12* and *che-13*, either individually or in combination with *skn-1* RNAi, on UbV-GFP turnover. In panels C and E, the scale bar corresponds to 400 μm. (**D**) The effect of RNAi knockdown of detoxification-associated proteins GST-24, UGT-39, UGT-48, CYP35A3, and CYP14A5 on UbV-GFP turnover. **(E)** FUdR impact on the cold survival of wild-type and *skn-1(mg570)* worms subjected to control, *pbs-6* and *gst-24* RNAi. Data was analyzed using two-way ANOVA and the significance levels obtained from the Šidák’s multiple comparisons test are indicated for the compared conditions (ns—not significant, ****—*P* ≤ 0.0001). At least 90 animals were scored in three independent biological replicates. In panels C and E FUdR treatment was carried out from the young adult stage for 40 hours.
(TIFF)

## References

[1] Hoppe T, Cohen E. Organismal Protein Homeostasis Mechanisms. Genetics. 2020;215(4):889–901. doi: 10.1534/genetics.120.301283 32759342 PMC7404231

[2] Kaushik S, Cuervo AM. Proteostasis and aging. Nat Med. 2015;21(12):1406–15. doi: 10.1038/nm.4001 26646497

[3] Hipp MS, Kasturi P, Hartl FU. The proteostasis network and its decline in ageing. Nat Rev Mol Cell Biol. 2019;20(7):421–35. doi: 10.1038/s41580-019-0101-y 30733602

[4] Kurtishi A, Rosen B, Patil KS, Alves GW, Moller SG. Cellular Proteostasis in Neurodegeneration. Mol Neurobiol. 2019;56(5):3676–89. doi: 10.1007/s12035-018-1334-z 30182337

[5] Antebi A. Regulation of longevity by the reproductive system. Exp Gerontol. 2013;48(7):596–602. doi: 10.1016/j.exger.2012.09.009 23063987 PMC3593982

[6] Kimble J, Crittenden SL. Controls of germline stem cells, entry into meiosis, and the sperm/oocyte decision in Caenorhabditis elegans. Annu Rev Cell Dev Biol. 2007;23:405–33. doi: 10.1146/annurev.cellbio.23.090506.123326 17506698

[7] Austin J, Kimble J. glp-1 is required in the germ line for regulation of the decision between mitosis and meiosis in C. elegans. Cell. 1987;51(4):589–99. doi: 10.1016/0092-8674(87)90128-0 3677168

[8] Grushko D, Boocholez H, Levine A, Cohen E. Temporal requirements of SKN-1/NRF as a regulator of lifespan and proteostasis in Caenorhabditis elegans. PLoS One. 2021;16(7):e0243522. doi: 10.1371/journal.pone.0243522 34197476 PMC8248617

[9] Chondrogianni N, Georgila K, Kourtis N, Tavernarakis N, Gonos ES. 20S proteasome activation promotes life span extension and resistance to proteotoxicity in Caenorhabditis elegans. FASEB J. 2015;29(2):611–22. doi: 10.1096/fj.14-252189 25395451 PMC4314225

[10] Berman JR, Kenyon C. Germ-cell loss extends C. elegans life span through regulation of DAF-16 by kri-1 and lipophilic-hormone signaling. Cell. 2006;124(5):1055–68. doi: 10.1016/j.cell.2006.01.039 16530050

[11] O’Brien D, Jones LM, Good S, Miles J, Vijayabaskar MS, Aston R, et al. A PQM-1-Mediated Response Triggers Transcellular Chaperone Signaling and Regulates Organismal Proteostasis. Cell Rep. 2018;23(13):3905–19. doi: 10.1016/j.celrep.2018.05.093 29949773 PMC6045774

[12] Lapierre LR, Gelino S, Melendez A, Hansen M. Autophagy and lipid metabolism coordinately modulate life span in germline-less C. elegans. Curr Biol. 2011;21(18):1507–14. doi: 10.1016/j.cub.2011.07.042 21906946 PMC3191188

[13] Shemesh N, Shai N, Ben-Zvi A. Germline stem cell arrest inhibits the collapse of somatic proteostasis early in Caenorhabditis elegans adulthood. Aging Cell. 2013;12(5):814–22. doi: 10.1111/acel.12110 23734734

[14] Steinbaugh MJ, Narasimhan SD, Robida-Stubbs S, Moronetti Mazzeo LE, Dreyfuss JM, Hourihan JM, et al. Lipid-mediated regulation of SKN-1/Nrf in response to germ cell absence. Elife. 2015;4. doi: 10.7554/eLife.07836 26196144 PMC4541496

[15] McColl G, Rogers AN, Alavez S, Hubbard AE, Melov S, Link CD, et al. Insulin-like signaling determines survival during stress via posttranscriptional mechanisms in C. elegans. Cell Metab. 2010;12(3):260–72. doi: 10.1016/j.cmet.2010.08.004 20816092 PMC2945254

[16] Gandhi S, Santelli J, Mitchell DH, Stiles JW, Sanadi DR. A simple method for maintaining large, aging populations of Caenorhabditis elegans. Mech Ageing Dev. 1980;12(2):137–50. doi: 10.1016/0047-6374(80)90090-1 6445025

[17] Van Raamsdonk JM, Hekimi S. FUdR causes a twofold increase in the lifespan of the mitochondrial mutant gas-1. Mech Ageing Dev. 2011;132(10):519–21. doi: 10.1016/j.mad.2011.08.006 21893079 PMC4074524

[18] Aitlhadj L, Sturzenbaum SR. The use of FUdR can cause prolonged longevity in mutant nematodes. Mech Ageing Dev. 2010;131(5):364–5. doi: 10.1016/j.mad.2010.03.002 20236608

[19] Anderson EN, Corkins ME, Li JC, Singh K, Parsons S, Tucey TM, et al. C. elegans lifespan extension by osmotic stress requires FUdR, base excision repair, FOXO, and sirtuins. Mech Ageing Dev. 2016;154:30–42. doi: 10.1016/j.mad.2016.01.004 26854551 PMC4789167

[20] Angeli S, Klang I, Sivapatham R, Mark K, Zucker D, Bhaumik D, et al. A DNA synthesis inhibitor is protective against proteotoxic stressors via modulation of fertility pathways in Caenorhabditis elegans. Aging (Albany NY). 2013;5(10):759–69. doi: 10.18632/aging.100605 24123581 PMC3838778

[21] Brunquell J, Bowers P, Westerheide SD. Fluorodeoxyuridine enhances the heat shock response and decreases polyglutamine aggregation in an HSF-1-dependent manner in Caenorhabditis elegans. Mech Ageing Dev. 2014;141–142:1–4. 10.1016/j.mad.2014.08.002 25168631

[22] Feldman N, Kosolapov L, Ben-Zvi A. Fluorodeoxyuridine improves Caenorhabditis elegans proteostasis independent of reproduction onset. PLoS One. 2014;9(1):e85964. doi: 10.1371/journal.pone.0085964 24465816 PMC3897603

[23] Davies SK, Leroi AM, Bundy JG. Fluorodeoxyuridine affects the identification of metabolic responses to daf-2 status in Caenorhabditis elegans. Mech Ageing Dev. 2012;133(1):46–9. doi: 10.1016/j.mad.2011.11.002 22116032

[24] Yoshioka A, Tanaka S, Hiraoka O, Koyama Y, Hirota Y, Ayusawa D, et al. Deoxyribonucleoside triphosphate imbalance. 5-Fluorodeoxyuridine-induced DNA double strand breaks in mouse FM3A cells and the mechanism of cell death. J Biol Chem. 1987;262(17):8235–41. 2954951

[25] Vermezovic J, Stergiou L, Hengartner MO, d’Adda di Fagagna F. Differential regulation of DNA damage response activation between somatic and germline cells in Caenorhabditis elegans. Cell Death Differ. 2012;19(11):1847–55. doi: 10.1038/cdd.2012.69 22705849 PMC3469062

[26] Lee HJ, Alirzayeva H, Koyuncu S, Rueber A, Noormohammadi A, Vilchez D. Cold temperature extends longevity and prevents disease-related protein aggregation through PA28gamma-induced proteasomes. Nat Aging. 2023;3(5):546–66. 37118550 10.1038/s43587-023-00383-4 PMC10191861

[27] Habacher C, Guo Y, Venz R, Kumari P, Neagu A, Gaidatzis D, et al. Ribonuclease-Mediated Control of Body Fat. Dev Cell. 2016;39(3):359–69. doi: 10.1016/j.devcel.2016.09.018 27746047

[28] Vilchez D, Morantte I, Liu Z, Douglas PM, Merkwirth C, Rodrigues AP, et al. RPN-6 determines C. elegans longevity under proteotoxic stress conditions. Nature. 2012;489(7415):263–8. doi: 10.1038/nature11315 22922647

[29] Lehrbach NJ, Ruvkun G. Proteasome dysfunction triggers activation of SKN-1A/Nrf1 by the aspartic protease DDI-1. Elife. 2016;5. doi: 10.7554/eLife.17721 27528192 PMC4987142

[30] Kahn NW, Rea SL, Moyle S, Kell A, Johnson TE. Proteasomal dysfunction activates the transcription factor SKN-1 and produces a selective oxidative-stress response in Caenorhabditis elegans. Biochem J. 2008;409(1):205–13. doi: 10.1042/BJ20070521 17714076

[31] Gu T, Orita S, Han M. Caenorhabditis elegans SUR-5, a novel but conserved protein, negatively regulates LET-60 Ras activity during vulval induction. Mol Cell Biol. 1998;18(8):4556–64. doi: 10.1128/MCB.18.8.4556 9671465 PMC109041

[32] Dantuma NP, Lindsten K, Glas R, Jellne M, Masucci MG. Short-lived green fluorescent proteins for quantifying ubiquitin/proteasome-dependent proteolysis in living cells. Nat Biotechnol. 2000;18(5):538–43. doi: 10.1038/75406 10802622

[33] Segref A, Torres S, Hoppe T. A screenable in vivo assay to study proteostasis networks in Caenorhabditis elegans. Genetics. 2011;187(4):1235–40. doi: 10.1534/genetics.111.126797 21288877 PMC3070531

[34] Matilainen O, Arpalahti L, Rantanen V, Hautaniemi S, Holmberg CI. Insulin/IGF-1 signaling regulates proteasome activity through the deubiquitinating enzyme UBH-4. Cell Rep. 2013;3(6):1980–95. 10.1016/j.celrep.2013.05.012 23770237

[35] Pispa J, Matilainen O, Holmberg CI. Tissue-specific effects of temperature on proteasome function. Cell Stress Chaperones. 2020;25(3):563–72. doi: 10.1007/s12192-020-01107-y 32306217 PMC7192876

[36] Jha S, Holmberg CI. Tissue-Specific Impact of Autophagy Genes on the Ubiquitin-Proteasome System in C. elegans. Cells. 2020;9(8). 10.3390/cells9081858 PMC7464313 32784405

[37] Lapierre LR, De Magalhaes Filho CD, McQuary PR, Chu CC, Visvikis O, Chang JT, et al. The TFEB orthologue HLH-30 regulates autophagy and modulates longevity in Caenorhabditis elegans. Nat Commun. 2013;4:2267. doi: 10.1038/ncomms3267 23925298 PMC3866206

[38] Melendez A, Levine B. Autophagy in C. elegans. WormBook. 2009:1–26. 10.1895/wormbook.1.147.1 19705512

[39] Galluzzi L, Baehrecke EH, Ballabio A, Boya P, Bravo-San Pedro JM, Cecconi F, et al. Molecular definitions of autophagy and related processes. EMBO J. 2017;36(13):1811–36. doi: 10.15252/embj.201796697 28596378 PMC5494474

[40] Kirkin V, Rogov VV. A Diversity of Selective Autophagy Receptors Determines the Specificity of the Autophagy Pathway. Mol Cell. 2019;76(2):268–85. doi: 10.1016/j.molcel.2019.09.005 31585693

[41] Wei W, Ruvkun G. Lysosomal activity regulates Caenorhabditis elegans mitochondrial dynamics through vitamin B12 metabolism. Proc Natl Acad Sci U S A. 2020;117(33):19970–81. doi: 10.1073/pnas.2008021117 32737159 PMC7443905

[42] Somers HM, Fuqua JH, Bonnet FXA, Rollins JA. Quantification of tissue-specific protein translation in whole C. elegans using O-propargyl-puromycin labeling and fluorescence microscopy. Cell Rep Methods. 2022;2(4):100203. 35497499 10.1016/j.crmeth.2022.100203 PMC9046455

[43] Garcia-Gonzalez AP, Ritter AD, Shrestha S, Andersen EC, Yilmaz LS, Walhout AJM. Bacterial Metabolism Affects the C. elegans Response to Cancer Chemotherapeutics. Cell. 2017;169(3):431–41 e8. 28431244 10.1016/j.cell.2017.03.046 PMC5484065

[44] Ghazi A, Henis-Korenblit S, Kenyon C. Regulation of Caenorhabditis elegans lifespan by a proteasomal E3 ligase complex. Proc Natl Acad Sci U S A. 2007;104(14):5947–52. doi: 10.1073/pnas.0700638104 17392428 PMC1851597

[45] Robinson JD, Powell JR. Long-term recovery from acute cold shock in Caenorhabditis elegans. BMC Cell Biol. 2016;17:2. doi: 10.1186/s12860-015-0079-z 26754108 PMC4709947

[46] Rogers AN, Chen D, McColl G, Czerwieniec G, Felkey K, Gibson BW, et al. Life span extension via eIF4G inhibition is mediated by posttranscriptional remodeling of stress response gene expression in C. elegans. Cell Metab. 2011;14(1):55–66. doi: 10.1016/j.cmet.2011.05.010 21723504 PMC3220185

[47] Howard AC, Rollins J, Snow S, Castor S, Rogers AN. Reducing translation through eIF4G/IFG-1 improves survival under ER stress that depends on heat shock factor HSF-1 in Caenorhabditis elegans. Aging Cell. 2016;15(6):1027–38. doi: 10.1111/acel.12516 27538368 PMC5114698

[48] Advani VM, Ivanov P. Translational Control under Stress: Reshaping the Translatome. Bioessays. 2019;41(5):e1900009. doi: 10.1002/bies.201900009 31026340 PMC6541386

[49] Roux PP, Topisirovic I. Signaling Pathways Involved in the Regulation of mRNA Translation. Mol Cell Biol. 2018;38(12). doi: 10.1128/MCB.00070-18 29610153 PMC5974435

[50] Seo K, Choi E, Lee D, Jeong DE, Jang SK, Lee SJ. Heat shock factor 1 mediates the longevity conferred by inhibition of TOR and insulin/IGF-1 signaling pathways in C. elegans. Aging Cell. 2013;12(6):1073–81. doi: 10.1111/acel.12140 23879233

[51] Lan J, Rollins JA, Zang X, Wu D, Zou L, Wang Z, et al. Translational Regulation of Non-autonomous Mitochondrial Stress Response Promotes Longevity. Cell Rep. 2019;28(4):1050–62 e6. doi: 10.1016/j.celrep.2019.06.078 31340143 PMC6684276

[52] Joshi KK, Matlack TL, Rongo C. Dopamine signaling promotes the xenobiotic stress response and protein homeostasis. EMBO J. 2016;35(17):1885–901. doi: 10.15252/embj.201592524 27261197 PMC5007557

[53] Guerrero GA, Derisbourg MJ, Mayr FA, Wester LE, Giorda M, Dinort JE, et al. NHR-8 and P-glycoproteins uncouple xenobiotic resistance from longevity in chemosensory C. elegans mutants. Elife. 2021;10. 10.7554/eLife.53174 PMC8460253 34448454

[54] Perkins LA, Hedgecock EM, Thomson JN, Culotti JG. Mutant sensory cilia in the nematode Caenorhabditis elegans. Dev Biol. 1986;117(2):456–87. doi: 10.1016/0012-1606(86)90314-3 2428682

[55] Li X, Matilainen O, Jin C, Glover-Cutter KM, Holmberg CI, Blackwell TK. Specific SKN-1/Nrf stress responses to perturbations in translation elongation and proteasome activity. PLoS Genet. 2011;7(6):e1002119. doi: 10.1371/journal.pgen.1002119 21695230 PMC3111486

[56] Lee BH, Lee MJ, Park S, Oh DC, Elsasser S, Chen PC, et al. Enhancement of proteasome activity by a small-molecule inhibitor of USP14. Nature. 2010;467(7312):179–84. doi: 10.1038/nature09299 20829789 PMC2939003

[57] Ermolaeva MA, Segref A, Dakhovnik A, Ou HL, Schneider JI, Utermohlen O, et al. DNA damage in germ cells induces an innate immune response that triggers systemic stress resistance. Nature. 2013;501(7467):416–20. doi: 10.1038/nature12452 23975097 PMC4120807

[58] Lee HJ, Noormohammadi A, Koyuncu S, Calculli G, Simic MS, Herholz M, et al. Prostaglandin signals from adult germ stem cells delay somatic aging of Caenorhabditis elegans. Nat Metab. 2019;1(8):790–810. doi: 10.1038/s42255-019-0097-9 31485561 PMC6726479

[59] Jiang W, Wei Y, Long Y, Owen A, Wang B, Wu X, et al. A genetic program mediates cold-warming response and promotes stress-induced phenoptosis in C. elegans. Elife. 2018;7. doi: 10.7554/eLife.35037 29664006 PMC5903861

[60] Murray P, Hayward SA, Govan GG, Gracey AY, Cossins AR. An explicit test of the phospholipid saturation hypothesis of acquired cold tolerance in Caenorhabditis elegans. Proc Natl Acad Sci U S A. 2007;104(13):5489–94. doi: 10.1073/pnas.0609590104 17369360 PMC1838478

[61] Anderson SM, Cheesman HK, Peterson ND, Salisbury JE, Soukas AA, Pukkila-Worley R. The fatty acid oleate is required for innate immune activation and pathogen defense in Caenorhabditis elegans. PLoS Pathog. 2019;15(6):e1007893. doi: 10.1371/journal.ppat.1007893 31206555 PMC6597122

[62] Pekec T, Lewandowski J, Komur AA, Sobanska D, Guo Y, Switonska-Kurkowska K, et al. Ferritin-mediated iron detoxification promotes hypothermia survival in Caenorhabditis elegans and murine neurons. Nat Commun. 2022;13(1):4883. doi: 10.1038/s41467-022-32500-z 35986016 PMC9391379

[63] Martineau CN, Kirienko NV, Pujol N. Innate immunity in C. elegans. Curr Top Dev Biol. 2021;144:309–51. doi: 10.1016/bs.ctdb.2020.12.007 33992157 PMC9175240

[64] O’Neil BH, Raftery L, Calvo BF, Chakravarthy AB, Ivanova A, Myers MO, et al. A phase I study of bortezomib in combination with standard 5-fluorouracil and external-beam radiation therapy for the treatment of locally advanced or metastatic rectal cancer. Clin Colorectal Cancer. 2010;9(2):119–25. doi: 10.3816/CCC.2010.n.017 20378507 PMC2893386

[65] Wang HF, Tong Y, Liu L, Liu HP, Li CD, Liu QR. The effects of bortezomib alone or in combination with 5-fluorouracil on proliferation and apoptosis of choriocarcinoma cells. Eur J Gynaecol Oncol. 2016;37(5):627–31. 29786999

[66] Brenner S. The genetics of Caenorhabditis elegans. Genetics. 1974;77(1):71–94. doi: 10.1093/genetics/77.1.71 4366476 PMC1213120

[67] Beydoun S, Choi HS, Dela-Cruz G, Kruempel J, Huang S, Bazopoulou D, et al. An alternative food source for metabolism and longevity studies in Caenorhabditis elegans. Commun Biol. 2021;4(1):258. doi: 10.1038/s42003-021-01764-4 33637830 PMC7910432

[68] Zou L, Wu D, Zang X, Wang Z, Wu Z, Chen D. Construction of a germline-specific RNAi tool in C. elegans. Sci Rep. 2019;9(1):2354. doi: 10.1038/s41598-019-38950-8 30787374 PMC6382888

[69] Qadota H, Inoue M, Hikita T, Koppen M, Hardin JD, Amano M, et al. Establishment of a tissue-specific RNAi system in C. elegans. Gene. 2007;400(1–2):166–73. doi: 10.1016/j.gene.2007.06.020 17681718 PMC3086655

[70] Espelt MV, Estevez AY, Yin X, Strange K. Oscillatory Ca2+ signaling in the isolated Caenorhabditis elegans intestine: role of the inositol-1,4,5-trisphosphate receptor and phospholipases C beta and gamma. J Gen Physiol. 2005;126(4):379–92. doi: 10.1085/jgp.200509355 16186564 PMC2266627

[71] Calixto A, Chelur D, Topalidou I, Chen X, Chalfie M. Enhanced neuronal RNAi in C. elegans using SID-1. Nat Methods. 2010;7(7):554–9. doi: 10.1038/nmeth.1463 20512143 PMC2894993

[72] Kamath RS, Ahringer J. Genome-wide RNAi screening in Caenorhabditis elegans. Methods. 2003;30(4):313–21. doi: 10.1016/s1046-2023(03)00050-1 12828945

[73] Arnold A, Rahman MM, Lee MC, Muehlhaeusser S, Katic I, Gaidatzis D, et al. Functional characterization of C. elegans Y-box-binding proteins reveals tissue-specific functions and a critical role in the formation of polysomes. Nucleic Acids Res. 2014;42(21):13353–69. doi: 10.1093/nar/gku1077 25378320 PMC4245946

[74] Schmidt EK, Clavarino G, Ceppi M, Pierre P. SUnSET, a nonradioactive method to monitor protein synthesis. Nat Methods. 2009;6(4):275–7. doi: 10.1038/nmeth.1314 19305406

[75] Doellinger J, Schneider A, Hoeller M, Lasch P. Sample Preparation by Easy Extraction and Digestion (SPEED)—A Universal, Rapid, and Detergent-free Protocol for Proteomics Based on Acid Extraction. Mol Cell Proteomics. 2020;19(1):209–22. doi: 10.1074/mcp.TIR119.001616 31754045 PMC6944244

[76] Myers SA, Rhoads A, Cocco AR, Peckner R, Haber AL, Schweitzer LD, et al. Streamlined Protocol for Deep Proteomic Profiling of FAC-sorted Cells and Its Application to Freshly Isolated Murine Immune Cells. Mol Cell Proteomics. 2019;18(5):995–1009. doi: 10.1074/mcp.RA118.001259 30792265 PMC6495249

[77] Tyanova S, Temu T, Cox J. The MaxQuant computational platform for mass spectrometry-based shotgun proteomics. Nat Protoc. 2016;11(12):2301–19. doi: 10.1038/nprot.2016.136 27809316

[78] Tyanova S, Temu T, Sinitcyn P, Carlson A, Hein MY, Geiger T, et al. The Perseus computational platform for comprehensive analysis of (prote)omics data. Nat Methods. 2016;13(9):731–40. doi: 10.1038/nmeth.3901 27348712

