# Supplementary material for: Floxuridine supports UPS independent of germline signaling and proteostasis regulators via involvement of detoxification in C. elegans
Source: PLoS Genet. 2024 Jul 31;20(7):e1011371. doi: 10.1371/journal.pgen.1011371 (PMC11318861; doi:10.1371/journal.pgen.1011371)
Supplement: S2 Table — (PDF) [file pgen.1011371.s002.pdf]

Table S2. Proteins up-regulated in wild-type, *glp-1(e2144)*, and *skn-1(mg570)* upon FUdR treatment with manually categorized functions.

Protein	Function
GST-24	Detofixication/innate immunity response
Y38H6C.15	Unknown
Y58A7A.3	Unknown
COL-41	Constituent of cuticle
F08G2.5	Unknown
F15D4.5	Unknown
NUMR-2	Unknown
FAR-3	Lipid regulation
COL-176	Constituent of cuticle
DUR-1	Unknown
PGP-8	Detofixication/innate immunity response
MAI-1	ATP regulation
M01G12.9	RNA regulation
PQN-22	Unknown
UGT-48	Detofixication/innate immunity response
Y45F10D.6	Unknown
DCT-17	Detofixication/innate immunity response
CYP-35A3	Detofixication/innate immunity response
F54B8.4	Detofixication/innate immunity response
UGT-29	Detofixication/innate immunity response
DAO-2	Unknown
CLEC-52	Detofixication/innate immunity response
UGT-39,UGT-38	Detofixication/innate immunity response
CYP-14A5	Detofixication/innate immunity response
CDH-7	Unknown
C14C6.5	Detofixication/innate immunity response
F10D2.10	Unknown
BAF-1	DNA regulation
VALV-1	Reproductive system regulation
SBDS-1	Ribosome regulation
ZK355.8	Unknown
ASD-2	RNA regulation
