# Supplementary material for: Floxuridine supports UPS independent of germline signaling and proteostasis regulators via involvement of detoxification in C. elegans
Source: PLoS Genet. 2024 Jul 31;20(7):e1011371. doi: 10.1371/journal.pgen.1011371 (PMC11318861; doi:10.1371/journal.pgen.1011371)
Supplement: S3 Table — (PDF) [file pgen.1011371.s003.pdf]

Table S3. The designated worm stages for specific RNAi treatments. Worms were considered as young adults 24 hours after L4 stage.

RNAi	Worm stage
pas-1	Young adult
pas-2	Young adult
pas-5	Young adult
pas-6	Young adult
pbs-2	Young adult
pbs-4	Young adult
pbs-5	Young adult
pbs-6	Young adult
rpn-1	Young adult
rpn-2	Young adult
rpn-3	Young adult
rpn-5	Young adult
rpn-6.1	Young adult
rpn-8	Young adult
rpn-9	Young adult
rpn-10	Young adult
rpn-11	Young adult
rpn-12	Young adult
rpt-1	Young adult
rpt-3	Young adult
rpt-4	Young adult
rpt-5	Young adult
rpt-6	Young adult
glp-1	L1
skn-1	L1
daf-16	L1
hsf-1	L1
pqm-1	L1
gst-24	L1
ugt-39	L1
ugt-48	L1
cyp-35A3	L1
cyp-14A5	L1
che-12	L1
che-13	L1
skn-1 + gst-24	L1
skn-1 + ugt-39	L1
skn-1 + ugt-48	L1
skn-1 + cyp35A3	L1
skn-1 + cyp14A5	L1
skn-1 + che-12	L1
skn-1 + che-13	L1
atg-1	Either young adults or L1
lgg-1	Young adults
fem-1	L1
let-363	L4
lmp-2	L4
hlh-30	L4
sqst	L4
chn-1	L4
glp-1 + pbs-5<br>*glp-1 + rpn-6.1*	L1 for UPS activity/ L4 for brood size assay and UPS activity
