# Supplementary figures and images for: Floxuridine supports UPS independent of germline signaling and proteostasis regulators via involvement of detoxification in C. elegans
Source: PLoS Genet. 2024 Jul 31;20(7):e1011371. doi: 10.1371/journal.pgen.1011371 (PMC11318861; doi:10.1371/journal.pgen.1011371)

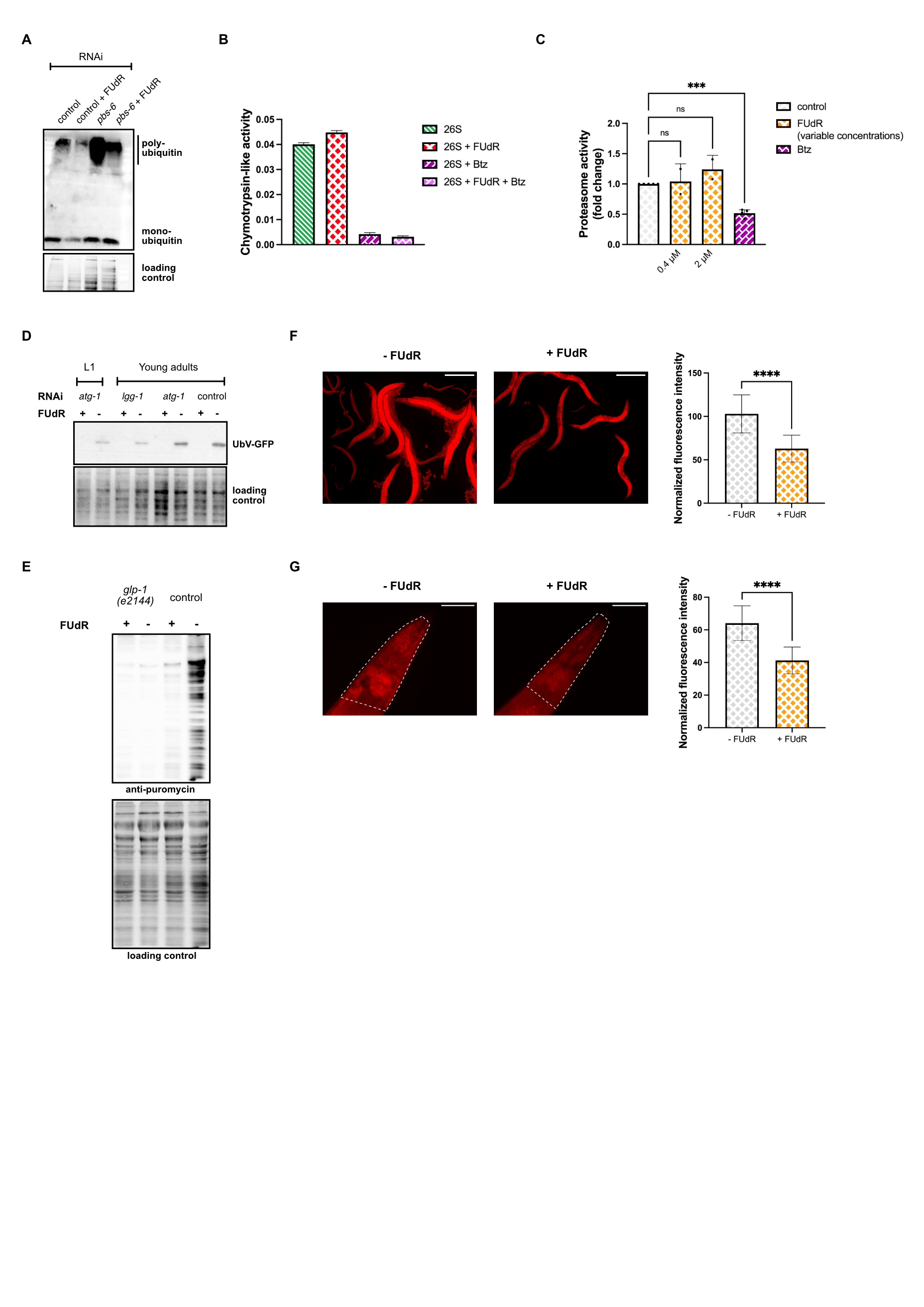

Supplement: S1 Fig — (A) Western blot showing the impact of FUdR on the accumulation of poly-ubiquitinated proteins in the presence of control RNAi or pbs-6 RNAi using anti-ubiquitin antibody. The No-Stain Protein Labeling Reagent was used to confirm equal protein loading. Silencing of pbs-6 and FUdR treatment was carried out from the young adult stage for 40 hours. (B) FUdR effect on the activity of purified human 26S proteasome as measured by chymotrypsin-like activity in the presence and absence of FUdR. Bortezomib (Btz) served as the negative control. Proteasome activity is represented as slopes obtained from kinetic measurements. The experiments were conducted thrice as separate biological replicates. (C) FUdR effect on chymotrypsin-like proteasome activity in HeLa cells. Cells were treated with final concentrations of 0.4 and 2 μM of FUdR and 10 nM bortezomib (Btz) as control for 6 hr. The assay was conducted by incubating the cells with 100 μl Proteasome Assay Loading Solution for 2 h as described in the methods. Results from three technical replicates were corrected for background by subtracting the fluorescence of the medium without cells and further normalized to dimethyl sulfoxide control. The graph shows the average values obtained from either two or four biological replicates for experiments that involve FUdR or Btz. (D) Western blot showing degradation of UbV-GFP in control, atg-1 RNAi (applied at either L1 or young adult stages), and lgg-1 RNAi (applied at the young adult stage) worms co-treated with bortezomib (Btz) in the presence or absence of FUdR, as depicted by using anti-GFP antibody. The No-Stain Protein Labeling Reagent was used to confirm equal protein loading. (E) Western blot showing global translation activity in wild-type and glp-1(e2144) worms, in the presence or absence of FUdR, as depicted by using the anti-puromycin antibody. The No-Stain Protein Labeling Reagent was used to confirm equal protein loading. (F) The level of RPS-6-mCherry was measured in ad [file pgen.1011371.s005.tiff]

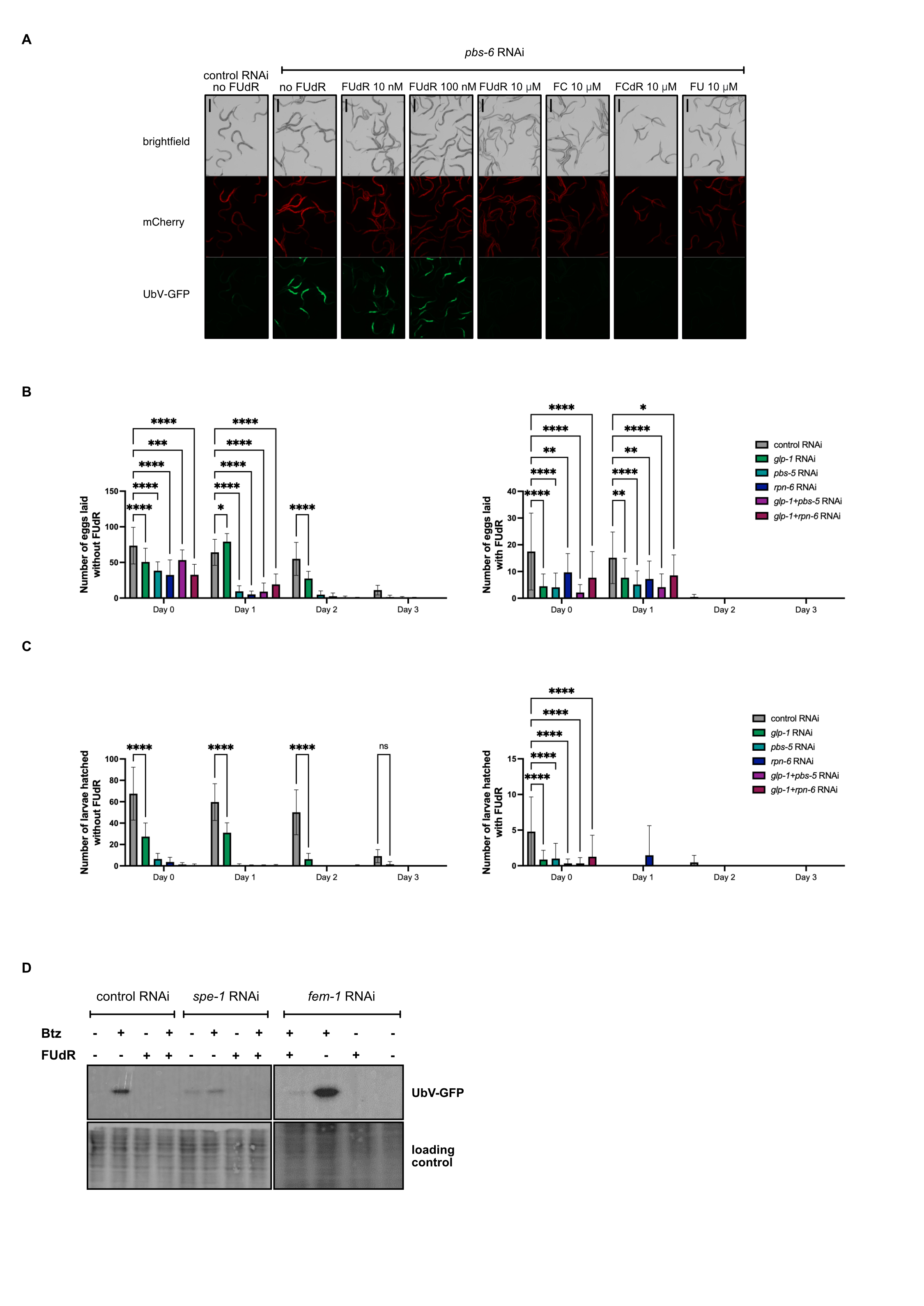

Supplement: S2 Fig — (A) The effect of various concentrations of FUdR and pyrimidine analogs, including 5-fluorouracil (FU), 5-fluorocytosine (FC), and 5-fluorodeoxycytidine (FCdR), on UbV-GFP turnover was assessed. The scale bar corresponds to 400 μm. FUdR treatment was applied at the young adult stage for 40 hours. (B) The graph shows the total number of eggs laid by UbV-GFP-expressing animals (2 animals per plate) in the presence or absence of FUdR, subjected to control, pbs-5, rpn-6.1, and double RNAi of (glp-1 + pbs-5) and (glp-1 + rpn-6.1) applied at the L4 stage. Data represent n = 30 from three independent biological repeats. Data was analyzed using two-way ANOVA and the significance levels obtained from the Šidák’s multiple comparisons test are indicated for the compared conditions (ns—not significant, *- P<0.05, **- P<0.01, ***-P<0.001, ****-P<0.0001). (C) The total number of larvae hatched by UbV-GFP expressing animals (2 animals per plate) was assessed in the presence or absence of FUdR, subjected to control, pbs-5, rpn-6.1 and double RNAi of (glp-1 + pbs-5) and (glp-1 + rpn-6.1) applied at the L4 stage. In panels B and C, FUdR treatment was initiated at the L4 stage. Data represent n = 30 from three independent biological repeats. Data was analyzed using two-way ANOVA and the significance levels obtained from the Šidák’s multiple comparisons test are indicated for the compared conditions (ns—not significant, ****-P<0.0001). (D) Western blot showing degradation of UbV-GFP upon RNAi depletion of SPE-1 and FEM-1, with or without bortezomib (Btz) or FUdR, detected using an anti-GFP antibody. Equal protein loading was confirmed with the No-Stain Protein Labeling Reagent. FUdR treatment was applied from the young adult stage for 40 hours. (TIFF) [file pgen.1011371.s006.tiff]

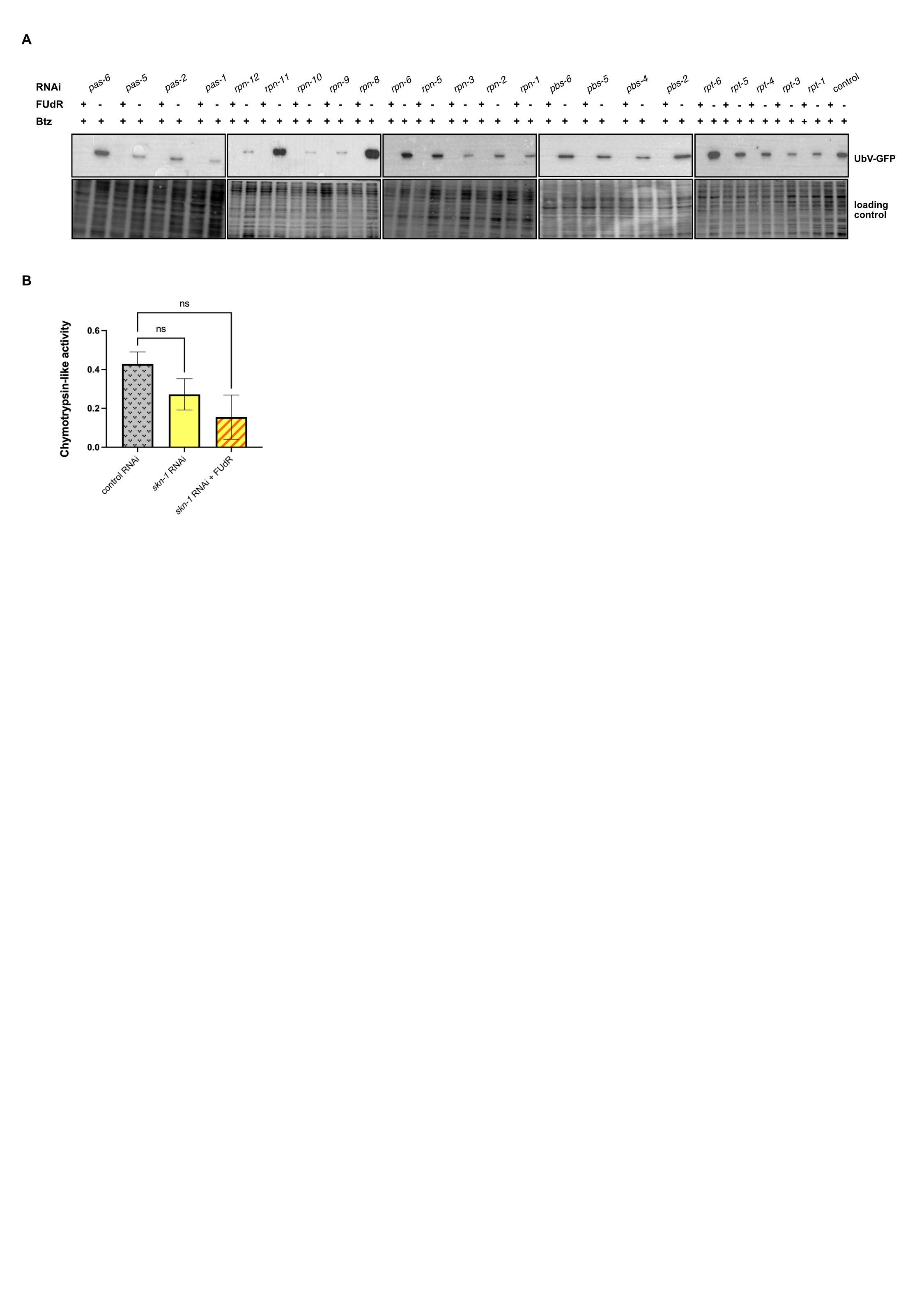

Supplement: S3 Fig — (A) Western blot showing the impact of FUdR on UbV-GFP reporter turnover upon depletion of various 26S subunits in control worms treated with bortezomib (Btz), detected using an anti-GFP antibody. The No-Stain Protein Labeling Reagent confirmed equal protein loading. Bortezomib and FUdR treatments were carried out from the young adult stage for 40 hours. (B) FUdR effect on proteasome activity, measured by chymotrypsin-like activity in wild-type worms subjected to control RNAi and skn-1 RNAi, with or without FUdR treatment (applied at the young adult stage for 40 hours). Proteasome activity is represented as slopes obtained from kinetic measurements. The experiments were conducted thrice as separate biological replicates, and significance levels (ns—not significant) were determined using an unpaired t-test with Welch’s correction. (TIFF) [file pgen.1011371.s007.tiff]

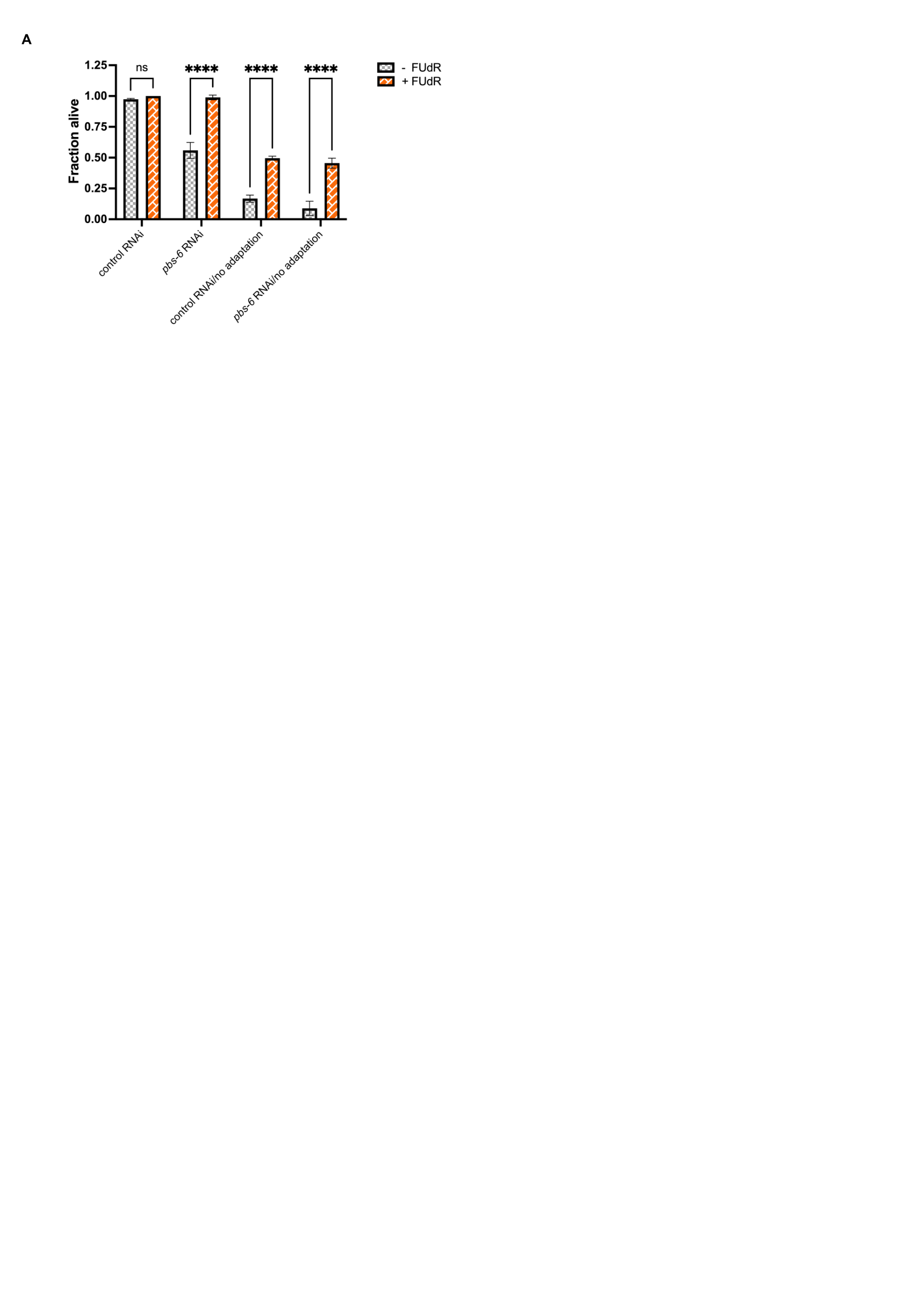

Supplement: S4 Fig — (A) The impact of FUdR on the cold survival of wild-type and pbs-6 knockdown worms, with or without a cold adaptation period. Data was analyzed using two-way ANOVA and the significance levels obtained from the Šidák’s multiple comparisons test are indicated for the compared conditions (ns—not significant, ****—P ≤ 0.0001). (TIFF) [file pgen.1011371.s008.tiff]

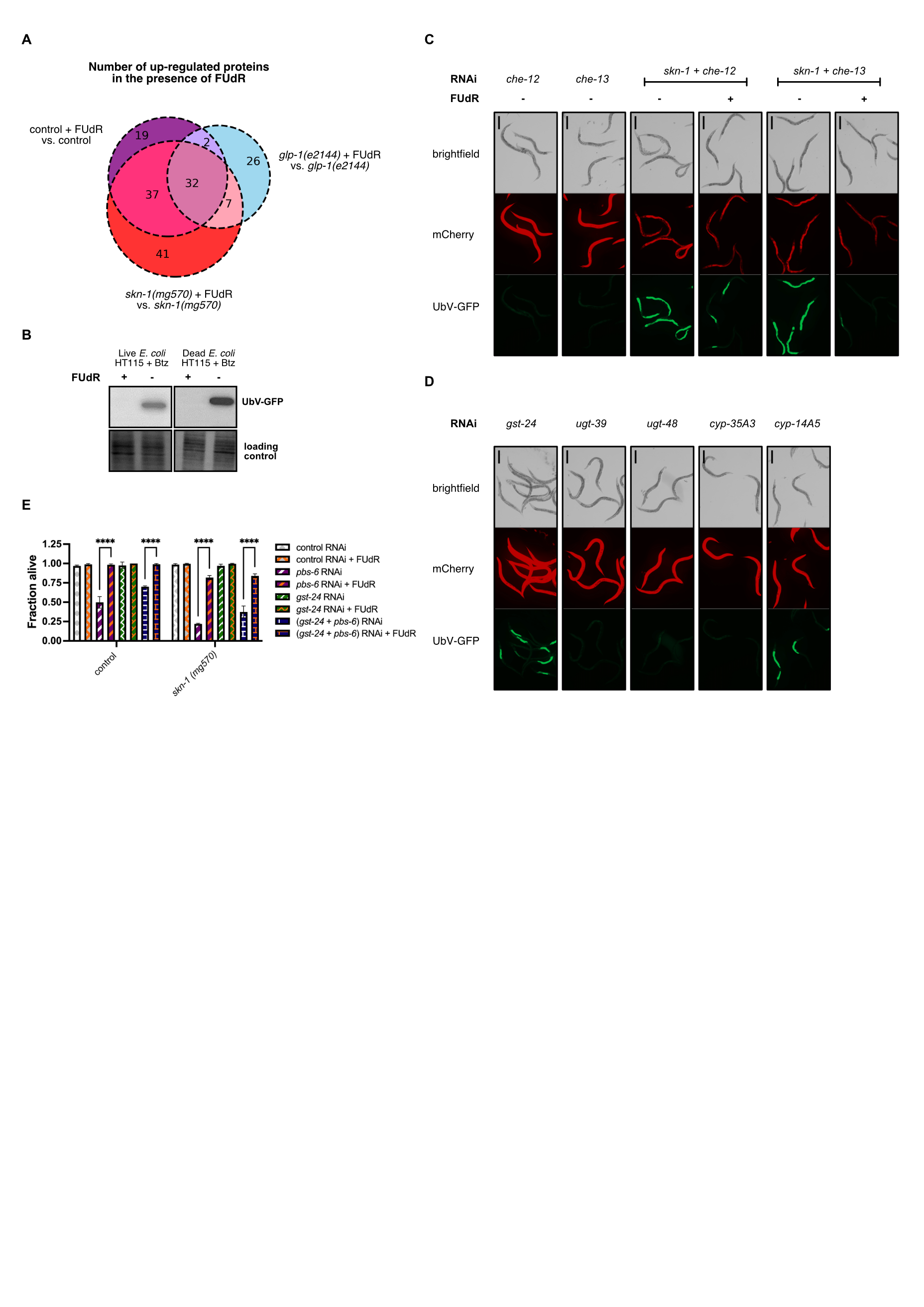

Supplement: S5 Fig — (A) Venn diagram showing the abundance of proteins up-regulated exclusively in the presence of FUdR in wild-type, glp-1(e2144) and skn-1 (mg570) worms. (B) Western blot showing the impact of bacterial viability on the UbV-GFP reporter turnover in the presence of bortezomib (Btz) and FUdR, as depicted by using anti-GFP antibody. The No-Stain Protein Labeling Reagent was used to confirm equal protein loading. (C) Impact of FUdR and RNAi knockdown of neuronal ciliary components che-12 and che-13, either individually or in combination with skn-1 RNAi, on UbV-GFP turnover. In panels C and E, the scale bar corresponds to 400 μm. (D) The effect of RNAi knockdown of detoxification-associated proteins GST-24, UGT-39, UGT-48, CYP35A3, and CYP14A5 on UbV-GFP turnover. (E) FUdR impact on the cold survival of wild-type and skn-1(mg570) worms subjected to control, pbs-6 and gst-24 RNAi. Data was analyzed using two-way ANOVA and the significance levels obtained from the Šidák’s multiple comparisons test are indicated for the compared conditions (ns—not significant, ****—P ≤ 0.0001). At least 90 animals were scored in three independent biological replicates. In panels C and E FUdR treatment was carried out from the young adult stage for 40 hours. (TIFF) [file pgen.1011371.s009.tiff]
